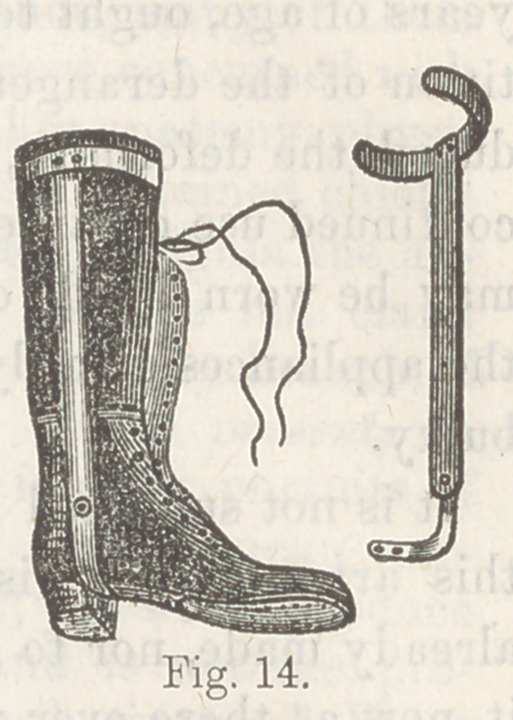# Report on Orthopedic Surgery

**Published:** 1864

**Authors:** David Prince

**Affiliations:** Jacksonville, Ill.


ARTICLE XXXIII.

### REPORT ON ORTHOPEDIC SURGERY.

BY DAVID PRINCE, M.D., Jacksonville, Ill.

Presented to the Illinois State Medical Society. May, 1864.

NOTE.—The verbal analysis of the report made to the Society covered portions of the whole ground of Orthopedic Surgery. By resolution of the Society, all reports were required to be furnished for publication by the first of July. It is impracticable, with other engagements, to complete the whole report in a satisfactory manner by that date. The portion embracing the group of deformities of the feet, known by the generic term TALIPES, is all that can appear in the Transactions for this year.

It is believed that the presentation to the profession, of the latest advances in this country, and in Europe, with the improvements introduced by the writer, will enable every practitioner to cure every uncomplicated case of congenital Talipes occurring in his own practice, if undertaken during the early months of infancy.

It is also believed that most cases, under fifteen years of age, are capable of successful treatment by patience, perseverance, and skill.

*Definition and Classification of the Genus, Species, and Varieties of Talipes.*

The term TALIPES, [Latin, *Talus* an ankle, and *pes* a foot,] has come to be adopted as a generic term for what is known as club-foot, reel-foot, and splay-foot, or flat-foot. The name expresses only a minor element of the deformity; the ankle, in some species, being not at all displaced or deformed, but this is of no great importance, since the technical signification has been agreed upon.

*Definition.*—A malposition or malformation of the foot, congenital or acquired, in which from some deviation at the ankle joint, or in a greater or less number of tarsal or tarso-metatarsal joints, the sole of the foot fails to apply to the ground in the natural position.

Of this genus there are six species:—

| | | | |
|---|---|---|---|
| Talipes | Equinus, | Talipes | Dorsalis, |
| " | Calcaneus, | " | Plantaris, |
| " | Varus, | " | Valgus. |

Of these species there are six possible secondary combinations or varieties, viz.:—

| | | | |
|---|---|---|---|
| Talipes | Equino Varus, | Talipes | Calcaneo Varus, |
| " | Equino Dorsalis, | " | Calcaneo Valgus, |
| " | Equino Valgus, | " | Calcaneo Plantaris. |

The conceptions of the tertiary combinations when once familiar, will also be simplified by classifying them thus:—

| | | | | | |
|---|---|---|---|---|---|
| Talipes | Equino | Varo Dorsalis, | Talipes | Calcaneo | Varo Dorsalis, |
| " | " | Valgo Plantaris, | " | " | Valgo Plantaris. |

*Talipes equinus*, is the term applied to that position which, by long continued voluntary elevation of the heel to compensate for several inches shortening of the limb, becomes not only habitual, but fixed by the permanent shortening of the triceps extensor pedis, and the adaptation of the ligaments to the habitual relations of the bones of the leg and tarsus. The habitual voluntary contraction of the triceps muscle, gastronemei, plantaris longus, and soleus, terminating in the tendo achilles, becomes permanent and involuntary; after which the muscular tissue changes its character; is absorbed or in part replaced by fat, while the white fibrous tissue investments become hypertrophied, converting the muscles into ligaments both in constitution and function. The result is a compensating deformity, and to attain the best possible compensation, bringing the phalanges as nearly as possible within the vertical line of pressure, the foot comes to be more than naturally arched by the contraction of the tibialis posticus, the peroneus longus, the flexor longus digitorum, upon the back of the leg, and the adductor pollices, the flexor brevis digitorum, the abductor minimi digiti, and the musculus accessorius with corresponding shortening of the plantar fascia under the foot. The action of the long and short flexors of the toes would curl them under the sole as the fingers are flexed upon the palm, if they were not kept out by the weight of the body upon the phalanges.

This makes the variety T. equino-dorsalis, which, in the confirmed state, is more common than either species unmixed. The deformity which has been described as originating in a voluntary attempt at compensation, may result from spasmodic contraction of one set of muscles, or paralysis of their antagonist.

*Talipes Calcaneus.*—A deformity in which the heel comes to the ground, and the anterior portion of the foot is drawn up by the disproportionate contraction of the tibiallis anticus, peroneus tertius, and extensor longus digitorum. This is a deformity so rare as only to be admitted as a possibility.

*Talipes Calcaneo Plantaris*, is a combination equally rare, in which the yielding is not chiefly in the triceps extensor pedis, but in the medio tarsal articulation between the astragalus and the calcaneum behind, and the scaphoid and cuboid before, with yielding to a smaller extent of the more anterior joints of the tarsus.

*Talipes Varus.*—This is the most common of all the species, whether congenital or acquired, and consists in the inversion and rotation of the anterior half of the tarsus which can, to a slight degree, be imitated by taking hold of the phalanges and metatarsus, and bending the foot in the direction in which the tebialis anticus would draw it. In making this twist, the calcaneum and astragalus will become adducted as in the position which a child will sometimes assume in standing upon the outer edge of the foot.

Attention has been called to a better anatomy of this deformity, by Mr. BARWELL, in his little book, entitled, "Club-Foot without Division of Tendons," in which he gives the appropriate name "medio tarsal articulation," to the articulation between the calcaneum and the cuboid on the outside, and between the astragalus and the scaphoid upon the inside. "This is the centre of the twist, which, in a delicate foot, can almost be imitated inward, while outward, or in the opposite direction, there is very little capability of a twist to bring down the inner side of the sole."

In this species there is no important contraction of the tri ceps, through the tendo achillis, or, in other words, a corresponding elevation of the heel. The heel is tilted over as if the hand were adducting the whole foot, by taking hold of the foot and pulling it inward. The inner or tibial edge of the foot is turned up, and the outer or fibular side turned down, and in the worst cases, carried in toward the opposite foot, so that the outer side of the dorsum of the foot comes to the ground. The sliding of the scaphoid outward upon the astragalus makes the former bone very prominent, receiving, with the cuboid and the anterior portion of the outer and lower edge of the calcaneum, the weight of the body, in standing and walking. The cuticle becomes unnaturally thickened, and between the integument and the bones, bursæ develop themselves as cushions to protect the bones from pressure in walking.

There is at first no transverse narrowing of the metatarsus and phalanges, but the pressure of walking gradually approximates the two borders of the metatarsus and phalanges; the fissure or concavity being in the plantar surface. The deformity appears to result from disproportionate contraction of the tibialis anticus, while the flexors and extensors are balanced, and the peronei muscles paralyzed. The tibialis posticus as-

Fig. 1. A Front View.

Fig. 2. A Back View.

sists in the inversion of the foot, so as to make the toes point toward the opposite foot.

This malposition is very well illustrated by the following cuts, representing the lower extremities of a gentleman fifty-two years of age, whose parents took him to Cincinnati, when an infant, to consult the best surgeons of that city. The parents were told that nothing could be done for the child.

*Talipes Equino Varus.*—This combination is the most common variety of talipes acquired subsequently to birth, and consists of disproportionate contraction of the triceps extensor pedis through the tendo achillis, elevating the heel and making a talipes equinus. The tibialis posticus tends to double the foot inward, while the tibialis anticus, at the same time, acts upon the inner edge of the foot, and elevates and rotates it, while the tibialis posticus, flexor longus digitorum, and the short flexors originating from the calcaneum, shorten the arch of the foot, making the compound expressed by the succession of terms, talipes equino-varo-dorsalis. Walking doubles the foot still more, antero posteriorly as well as transversely, almost completely turning it up side down, giving the gait a much worse hobble than that of simple varus, and presenting a complicated deformity, requiring apparatus equal to the versatility of the hand for its successful treatment.

*Talipes Dorsalis.*—An unnatural elevation of the arch of the foot, by a change in the medio-tarsal articulation, or the tarso metatarsal articulation, or in all combined. This condition has already been noticed in combination, in T. equino dorsalis, and T. equino varo dorsalis. It may exist as an uncombined deformity, either as a natural development; as the result of disease, or injury, or as an artificial production. The shape of the foot produced by the Chinese shoe, is a shortening of its length and a humping up of the instep, making a stumped appearance, a talipes dorsalis.

It may also result from a partial dislocation, breaking up the ligamentous fastenings on the dorsum of the foot, and permitting a shortening of the base of the tarso-metatarsal arch. This once occurred under the observation of the writer—a young man falling twenty feet from a tree, and dislocating the tarso-metatarsal articulation of both feet. The deformity was never completely reduced, and the tarso-metatarsal joints remained permanently elevated, requiring shoes to be made according to special measurements.

*Talipes Plantaris.*—Flat foot, the condition in which the sole comes to the ground in all parts; there being little or no arch. This is the natural condition in a portion of the negro race, and is often the result of want of action of the tibialis anticus, and T. posticus, resulting in elongation of the plantar facia, from too great tension of it. In feeble children it comes from premature walking.

*Talipes Valgus.*—The condition in which the anterior half of the foot is carried outward in the direction opposite to that of T. varus. The tibialis anticus and tibialis posticus fail, and the peroneus longus and P. brevis, passing behind the external malleolus, pull upon the outer side of the foot and evert it. At the same time the peroneus tertius passing down in front of the ext. malleolus elevates the outer side of the foot, and tilts the astragalus and calcaneum outward in the opposite direction to that taken in T. varus.

The following cut illustrates this species, which is rarely met with, without complication.

Fig. 3.

The figure is taken from the cast of the foot of a gentleman living in Boston. The cast is kept by Messrs. TIEMANN & Co., Surg. Inst. Makers, N. Y., for the purpose of making upon it the apparatus which aids him in walking.

The figure is seen from behind and on the inner side.

It will be noticed that this is a simple T. valgus, without any flattening of the arch of the foot, to make the species plantaris. The more common development is

*Talipes Valgo Plantaris.*—The condition in which the anterior half of the foot is carried outward and upward, bringing the inner side of the tarsus to the ground, while the arch of the foot is lost by the relaxation of the muscles, ligaments, and fascia which sustain it. As the deformity progresses, the extension, or downward projection of the medio-tarsal joint, permits the metatarsus to rise altogether from the ground, by the action of the peroneus tertius, leaving the weight to come altogether upon the tarsus. This extreme perversion, however, constituting a talipes calcaneo-valgo plantaris, is rarely attained. When existing it must arise from the action of the extensor longus digitorum acting in concert with the peronei muscles, or more commonly from paralysis of the opposing flexor and adduct or muscles.

*Talipes Calcaneo Varus* is only a possible variety, resulting from disproportionate action of the tibialis anticus, and T. posticus, the triceps extensor pedis being paralyzed so as to permit the long flexors to elevate the metatarsus, while the heel remains depressed.

This classification may seem unnecessary, but it is the shortest way of describing the great variety of deformities classed under the generic term Talipes. Having once become familiar with terms, they will ever afterwards convey definite ideas, not only of the forms but of the muscular contractions which must be concerned in producing and perpetuating them.

A clear idea of these conditions will lead to a rational interpretation of the indications of treatment whether preventive or curative.

Similar directions, from the normal form of the hand, should receive a similar classification, only that their rareness makes it unnecessary. Their pathology is doubtless the same, whether con. or post. genital, depending upon paralysis of one class of muscles, or overaction of their antagonists, or both combined; or more rarely, some accidental injury, resulting in partial dislocations ending in permanent deformity, or from the contraction of the cicatrices of burns or ulcers.

*Complications.*

1. The complications may be congenital or acquired, absence or diminution of one or more bones, implying the impossibility of complete restoration of the form and functions of the foot, though great improvement may, in some cases, be effected by treatment.

2. Anchylosis of one or more joints from fractures or wounds, nearly or quite hopeless of benefit from subsequent treatment.

3. Anchylosis from arthritic or periosteal inflammation, in which the treatment is chiefly preventive, by substituting, before it is too late, passive motion for absolute rest of the parts in relation to each other.

4. Contraction of cutaneous cicatrices from burns, ulcers, or wounds. The treatment should be preventive, for confirmed deformity, from these sources, is extremely difficult to overcome.

5. Rheumatism, producing talipes, or simply attacking a taliped, requiring the abatement of the rheumatism in addition to whatever else may be done.

6. Corns and bunions requiring nice adaptation of shoes where, from the age of the patient, they cannot be cured by restoring the foot to its proper form.

7. Absence or deficiency of toes.

8. Supernumerary toes which may be cut off.

9. Deviation of the forms and directions of the toes from fractures, wounds, arthritic, or periosteal inflammation, the contractions of cicatrices from burns or other injuries, from faulty shoes, from pressure of the weight of the body, or from paralysis of muscles. These deviations are sometimes incapable of remedy except by amputation of the offending toes.

*Causes and Nature of Talipes and Allied Deformities.*

The nice adjustment of forces by which typical symmetry is produced and maintained, in all organized growth, only needs to be contemplated to secure admiration.

The exceptional deformities, proving the possibility of imperfect adjustment of these forces, or of the occurrence of accidental impediments to their exercise, only excites our attention all the more, to the nice balance observed in the ordinary working of the law of development.

In individual failures of this organic law of symmetry, the question will arise as to the modes of deviation:—

1. Whether from excessive nutrition, analogous to that which secures the disproportionate growth in parts which are brought to perform compensating functions, as a leg or a kidney, which from the impairment or destruction of the opposite, is invited to perform more than its natural part.

2. From deficient nutrition direct, from the obstruction of the bloodvessels which supply it, or indirect, from failure of nervous supply to the capillaries of a part, failing to open them to the supply of blood, or from accidental or artificial quietude, analogous to that of muscles closely confined in splints and bandages, while a fractured bone is uniting.

3. From accidental positions, widely varying from those which are usual and which act to produce deformities, like the forces which are afterward employed to remove them. By this means, some tendons may be forced to grow too long, and others permitted to become too much shortened, while the bones which become inordinately compressed take the shapes which the altered forces tend to give them.

4. From some observations made by CRUVEILHIER, this careful pathological anatomist came to the conclusion that position of the foot, within the uterus, was often a cause of talipes.

As a moderate talipes varus is the ordinary position of the foot within the uterus, this deformity can hardly be explained upon this hypothesis; but a talipes valgus might possibly be produced by an eversion of the foot from the pull of the umbilical cord accidentally entangled around it.

Twisting and displacements and spontaneous dislocations of the knee-joint, of the hip-joint, and of the shoulder-joint, can sometimes be most plausibly explained upon this supposition.

5. From the occurrence of causes which directly compress, or partially or completely cut off, portions of the developing limbs; portions of the liquor amnii unusually condensed or solidified into sheets or shreds, may produce deep fissures in parts upon which they press; or they may completely amputate the included parts. The peculiar deformities constituting the genus talipes can hardly be explained by reference to this class of causes. Spontaneous amputations doubtless often owe their occurrence to this cause.

6. From disease directly resulting in the death of the parts affected. The writer has in his possession an aborted fœtus of four months, which exhibits gangrene of one upper extremity, including the shoulder. If this fœtus had lived, there would have been the birth of a one-armed child. Spontaneous amputations are sometimes produced by this cause, but talipes cannot be thus explained.

7. From the union of parts of two or more individuals, resulting in redundancy of number. This is the explanation of a great variety of monstrosities, but it does not apply to talipes.

8. From an influence existing in the germinal origin of the individual, like that which determines the color of the skin, the family likeness of features, and the temperament. It is thus, that in some families there is a perpetuation, through several generations, of five fingers upon the hand and six toes upon the foot, the deficiency of a thumb or a redundant one.

Though several cases of talipes sometimes occur in one family, and in rare cases it may be repeated in the next generation, the cases are too few to favor this explanation of its occurrence. Causes acting upon the innervation of the fœtus, subsequent to the formation of the type of the individual, constitute a more probable explanation.

9. From causes set in operation through physical and mental influences of the mother. As an example of physical influence, one of the common expedients for distinguishing pregnancy from enlargements within the abdomen from other causes, is to place the hand, previously reduced in temperature, upon the mother's abdomen, to excite a convulsive movement in the fœtus. This movement may be stimulated by the compression made by the sudden tension of the abdominial muscles induced by the cold application.

On the other hand, great physical exertion, and the occurrence of grave disease affecting the constitution of the circulating fluids, are followed by diminution or cessation of the fœtal movements, as if from some diminution of the fitness of the blood to afford to the fœtus the highest activity of nutrition. The death of the fœtus, and its expulsion is a frequent occurrence under these circumstances.

That deformities should sometimes arise from this impaired or perverted nutrition, is as probable, as that similar disturbances should, after birth, produce local congestions and inflammation, or convulsions and paralysis; some constitutional tendency, previously induced, determining the location and character of the diseased action.

Protracted mental depression, the indulgence of ungoverned anger, hate, or revenge, impairing digestion, are supposed to be unfavorable to the best development of the fœtus, while the cheerful and joyous emotions are invited as most favorable.

With the shock from the sight of a repulsive object, the mother feels a convulsive movement of the fœtus, followed by a diminution of the habitual movements, and her attention is afterward anxiously fixed upon her own sensations and those produced in her by the fœtus.

Derangement of the digestion of the mother, and the consequent impairment of the healthy and nutritive qualities of her blood, which is the source of nutriment to the fœtus, often exist for a longer or shorter period, and deformities sometimes follow, but at the birth, the mother's fears are generally found to have been needless, as a perfect form occupies the place of the dreaded deformity.

In the few cases that do occur, there are, in exceptional instances, striking resemblances to some object seen by the mother during pregnancy; but upon close scrutiny of the deformities they are found to belong to classes of excessive, deficient, perverted, or arrested development already referred to, from the various causes classified; and these resemblances are too few, in comparison with the whole number, to be worthy of any other explanation than that of coincidence. We all know how a striking coincidence takes more hold upon the mind than many discrepancies. The adoption, early in the civilization of all nations, of the theory of the direct production of special deformtieis through the images impressed upon the mind of the mother, is probably thus best explained.

The deformities arising from spasm and paralysis are more frequent in the lower extremities, from the more feeble, more easily deranged, and less easily restored innervation of those parts. They are, therefore, more often seen in the streets, and from the awkward movements in walking, they are more repulsive than deformities of the upper extremities, which need not be made conspicuous in public places.

The late development and comparatively low innervation of the inferior half of the fœtus might be expected to result in the existence at birth of a greater number of deformities, produced by nervous derangement, in the inferior than in the superior half of the body. From this physiological order of development, as well as upon the hypothesis of coincidence, therefore, a mother who is shocked at the sight of a lame leg is more likely to have a child affected with talipes, than with a corresponding deformity of the hand, the deteriorating influence of the nervous impression upon the blood being more likely to result in spasmodic or paralytic affections of the lower than of the upper extremities of the fœtus.

As the varus species of congenital talipes are similar to the corresponding deformities developed subsequently to birth, from derangements of innervation, it is fair to infer, that in most cases, a similar derangement of innervation has existed during fœtal life. This conjecture is rendered more probable by dissection, which shows that the bones of the tarsus have their proper forms until they are afterward slightly changed in figure, by the great pressure to which they are subjected in walking. This change is much less than a superficial glance would lead one to suppose, there being nowhere a complete dislocation, but only a sliding a little further than the normal length of the ligaments permits.

The following figure, taken from "LITTLE, on the Nature and Treatment of the Deformities of the Human Frame," sufficiently illustrates this point:—

The relative importance of paralysis and spasm, in the produc-

Fig. 4.

tion of this and other deformities, will be differently appreciated by different minds, standing in opposite positions. The following quotation from BAUER, (1) representing the older pathology, and from BARWELL, (2) representing the newer, illustrate this point.

Dr. BAUER (p. 12) thinks contraction of the sural muscles, the muscles ending in the tendo achillis, generally the chief cause of the extension of the foot in talipes equinus. He makes no account of the doubling up of the foot at the medio tarsal articulation, so carefully explained by LITTLE and BARWELL, and, equally with BARWELL, omits to mention the calcaneo-metatarsal and calcaneo-phalangeal muscles, as elements in the etiology.

Referring the disease to the shortened muscles, he says, "As a general thing, the contracted muscles have lost all susceptibility of being acted upon by the galvanic current, yet their powerful extension gives rise to unbearable pain. This fact seems to demonstrate that the muscular structure is in a state of contraction to the extent of its capacity, or the substituted tissue is void of all contractile" (expansive) "power. It is certain that innervation has not been entirely lost, while pain can be provoked by extension."

In the conditions referred to in this paragraph, the occurrence of pain may, perhaps, be better explained by bearing in mind that the muscles concerned have, for the time, acquired the conditions of ligaments.

(1) Lectures on Orthopedic Surgery, by LOUIS BAUER, M.D. Lindsay & Blakiston, Phila., 1864.

(2) The Treatment of Club-Foot without the Division of Tendons, by Mr. RICHARD BARWELL, &c. London, 1863.

We know well enough, that ligaments are susceptible of acute pain when overstretched. When a muscle, therefore, which has lost its function from loss, change, or paralysis of its muscular substance, is pulled further than its investments of white fibrous tissue will permit, without injury to its habitual physical condition, it is in close analogy with an overstretched ligament, and it should be the seat of pain, the same as if it had originally been a ligament.

The following additional quotation is a further illustration of the spasmodic pathology:—

"After the division of tendons, many months may elapse before the galvanic current makes any impression, and in some instances the contractibility of the muscles is gone forever."

If the division of tendons is all that is done, the shortening ought to go on still more. It is, probably, the subsequent movements, effected in the course of the treatment, that restore the susceptibility of the galvanic current.

Dr. Bauer finds an advocate for the doctrine of tonic spasm, as the cause of talipes equinus, in Dr. Joseph Pancoast,* of Philadelphia, who thinks, that of the three muscles uniting to make the tendo achillis, only the soleus is inordinately contracted, and accordingly, he only divides the soleus in the treatment. This is done by passing the bistoury under the gastroc nemeus, and cutting the soleus just as it becomes tendinous

* Dr. Joseph Pancoast, of Philadelphia, claims that the elevation of the heel in talipes equinus is owing to the contraction of the soleus while the gastrocnemeus remains flaccid; and he accordingly divides the soleus muscle by passing a knife in under the gastrocnemeus, instead of the usual easy method of dividing the tendo achillis.

It is found in any confirmed case of talipes equinus or T. equino varus, that the soleus is rigid and incapable of extension while the gastrocnemeus is yielding. Dr. Pancoast is, therefore, of opinion that the soleus is the autho f the mischief. The fact has another explanation. When a muscle contrac with such power that its antagonists cannot extend it, the more powerful mus cle soon becomes inextensible, and it settles into the function of a ligament, holding firmly the points to which it is attached, the muscular tissue gradually becoming atrophied, and while the size of the muscle diminishes its hardnes increases.

This is the state of the soleus in extreme talipes equinus. The upper end i attached to the tibia and fibula; and when the calcaneum is elevated as far a its ligaments and bony connexions will permit, the soleus can contract no furand unites with the gastroc nemeus; the edge of the knife being carried toward the bones for this purpose.

It would be wrong, however, to leave the reader with the impression that Dr. BAUER considers spasm the uniform cause of talipes, and the following question, from p. 19, of this book, will do him justice in this respect:—

"After mature deliberation, we have come to the conclusion, that the cause in congenital as well as acquired club-foot, is preëminently defective innervation; and there is truly no reason why derangements in the nervous system should not take place in the fœtus as well as in the new-born child. In club-foot, the tibial nerve is the bearer of the difficulty, as must be inferred from the experiments of BONNET."

* * * * "All forms of varus are caused by either muscular contraction or motor paralysis, and the individual bones of the foot yield only so much in their respective positions, as they are forced to do, by the abnormal muscular traction, and the superincumbent weight of the body. Being held for some time, and acted upon in the preternatural position, they gradually mould themselves accordingly, and become consequently malformed."

In the opposite pathological view, it is claimed by that careful observer, Mr. RICHARD BARWELL, that it is not usually spasm of the stronger, but paralysis of the weaker muscles, which lies at the foundation of the deformity, and in support of this view he refers to the common experience, that in talipes the temperature is generally low, while in spasm it is generally high.

ther, and if not lengthened by an opposing power, it at length becomes hard and unyielding. This result is prevented in the gastrocnemeus by its attachment to the femur, whose movements keep this muscle active and extensible. After the soleus has become rigid from *immobility*, the gastrocnemeus continues to have *mobility*, and, therefore, it preserves its extensibility. It is not that it draws less, but that it never acquires a stationary contraction, and, therefore, never comes into an unyielding condition.

Disproportionate weakness of the flexors of the foot, with anchylosis of the knee-joint, would probably result in equal extreme contraction, and consequent rigidity of gastrocnemeus and soleus alike.

This explanation entirely destroys the value of Dr. PANCOAST's method of dividing the soleus instead of dividing the tendo achillis, in permanent elevations of the heel.

"Infants, as is well known, are subject to convulsions; and it is averred that sometimes one or more muscles, which have, during the attack, drawn the limb into malposture, do not recover from the contraction, but continue to keep the limb distorted. * * The state should be one of persistent unvarying spasm, powerful enough to overcome the antagonistic healthy muscles, and permanent enough to produce lasting change of form. Such condition does not only never come under our notice, but it is, I believe, pathologically impossible. There are, no doubt, a few cases of peculiar paralysis of the voluntary power over the muscles, while the excito-motory function continues; and in the spasm of the whole set, the strongest organ will of course predominate. Voluntary power is as much used to control as to excite. The paralysis of this power is as much evidenced by violent and uncontrollable spasm, as by incapability of subordinate movement. In my experience, such state seldom continues long, unless there be cerebral disease or deficiency, but terminates, within a limited period, in death or complete recovery, or in simple paralysis in one set, and perfect restoration of power in another set of muscles." * * * * "Laryngismus stridulus, or false croup, is attributed, by some, to spasm of certain muscles; while by other authorities, and I believe with more reason, it is considered as paralysis of a different pair. Let it be observed, also, that the squint which may come and go with other symptoms of brain mischief, or may be permanent affection, is certainly to be more rationally regarded as want of action in the outer rectus, which appropriates the whole of one nerve, (the sixth), than as spasm of the inner rectus, whose nerve supplies four other muscles of the eye and appendages. Certain, also it is, that some congenital deficiencies of the nervous system, whereof club-foot and club-hand are pretty constant accompaniments, as acephalosis, &c., &c., may, indeed must, produce paralysis, but there is no evident connexion between such deformity and spasm." p. 23.

"Altogether, there can be no doubt that paralysis is very much more frequently the cause of club-foot than the opposite condition. * * * The morbid contraction of a muscle, or set of muscles, is hardly ever violent enough, or persistent enough, to cause a permanent alteration in the shape of the foot, where the opposers remain active."

"The muscles, while healthy, are always kept at a certain degree of tension by tonic contraction, but when any one organ or set of organs has lost its power, the opposers draw the limb in the opposite direction, by virtue of that constant and elastic sort of force. For a long time after the commencement of the paralysis, there is nothing whatever wrong with the active muscles, but after they have been allowed to become thus short, for a certain period, they begin to adapt themselves to the shortened condition, and still further contracting, as they meet with no resistance, determine at last changes of form in other structures, and so produce permanent deformity." The clearness with which the points are here made, justifies the length of the quotations.

*Treatment.*

It is believed that a careful consideration of the nature and pathology of the different varieties of talipes, as explained in the preceding pages, will afford the foundation for clear ideas of the indications of treatment, whether preventive or curative. The plans and expedients for meeting these indications are now the earnest study of those interested in this branch of surgery. No words of mine can be more appropriate than those of BARWELL. (p. 25.)

"It is not to be imagined, that when the limb has yielded in the direction of the healthy muscles, the sickly ones can recover sufficiently quickly or entirely to restore, by their unassisted might, the proper balance of the foot. The weakened muscles want assistance; and the way to render this, in the manner which shall best aid them to overcome the deformity, and to recover from the paralyzed or enfeebled condition, is the problem which surgeons should endeavor to solve."

It is one of the points showing the impossibility of practically and completely separating Medicine from Surgery, and the different branches of Surgery from each other, that in these cases of paralysis, previous to the occurrence of obvious deformity, the disease would be said to be in the department of Medicine, though mechanical or chirurgical means are necessary to prevent the occurrence of deformity; and afterwards, when the deformity places the disease fairly in the department of Surgery, the best period for surgical treatment has been allowed to pass by: because the case was in the department of Medicine.

The Physician must study Surgery, and the Surgeon must study Medicine.

Whoever has examined a case of club-foot, by taking hold of it with his hands, may have thought, that if he only had some machine that would take hold of the foot as firmly, and yet as tenderly as does the hand, without relinquishing its grasp, and yet pulling yieldingly but persistently and without tiring out, he could cure any case. The defect of every metallic apparatus is, that while it grasps the foot firmly enough, it pulls unyieldingly, without that distribution of force among all the distorted joints, which is effected by the hand. They are, most of them, intended to act chiefly upon the tibio tarsal joint, while the most careless inspection of any species of talipes, except one of simple talipes equinus, will show that the distortion of this joint is a minor element in the case.

That an adequate substitute for the hand is a desideratum not yet furnished to the public, is sufficiently proved by the words of Dr. Bauer. (p. 23.)

"There is no mechanical apparatus, however ingeniously constructed, which could be substituted for the hand, in the treatment of talipes, with any approximate degree of efficiency. In fact, if we could without interruption, employ the hand, as a mechanical agent, we should relieve most obstinate forms of talipes, which *too frequently* withstand our mechanical appliances." This is an estimate of the importance of some substitute for the hand, with an expression of hopelessness as to its attainment.

On the other hand, Dr. Gross, in his great work on surgery, v. II, p. 1011, is well enough satisfied with our present attainments in the art, neither desiring nor expecting any improvements. He says, "It is perhaps not going too far to affirm that these topics" (club-foot) "are as well understood now as they ever will be."

Dr. BAUER again places this estimate upon our present attainments, (p. 28), "They" (mechanical appliances) "possess no curative virtues, but retain the foot in the position in which tenotomy and the acting hand left it."

It is believed that, in the course of these pages, a process will be explained, which is a pretty adequate substitute for the hand.

The earlier experimenters in this art seem to have relied chiefly upon wood and iron, as substitutes for the hand; but so generally did they occasion ulcerations of prominent parts, that the art made no important progress until the introduction of subcutaneous section of tendons, by STROMEYER, in 1831. In a large proportion of the cases of talipes, including all the species equinus, the division of the tendo achillis, permits an immediate improvement in the position of the foot, and facilitates the further reduction of the distortion of the joints of the tarsus. This tendon had been cut at various times before STROMEYER, by making an open wound; but this procedure could never be generally adopted. Dr. H. G. DAVIS, in his report on deformities, in the Transactions of the National Medical Association, 1863, quotes ISAAC MINCIUS as having divided it in 1685; THELLENIUS in 1784; SARTORIUS in 1806; MECHAELIS in 1809; DELPECH in 1816; but none of these men could think of so simple an expedient as passing in a small knife at a point distant from the tendon, and so dividing it, that the incision through the skin should heal without suppuration. It is commonly recommended, with a sharp pointed bistoury, to puncture the skin upon the inner or tibial side of the tendon opposite the internal malleolus, or higher if the heel is very much elevated, and having withdrawn this to pass a probe-pointed bistoury between the tendon and the tibia, and while the tendon is made very tense by the hand of an assistant holding the foot, to cut the tendon by pressing the fingers upon it, thus crowding it upon the knife. If any shreds remain undivided, the fact is known by the failure of the heel to come down, and the bistoury is again partially withdrawn and passed under them, when they are divided by the same process by which the main portion of the tendon was cut. The reason for passing the knife on the tibial side of the tendon, is the less danger of wounding, by the point of the knife, the posterior tibial artery, which lies upon the inner side, and the same reason exists for cutting towards the skin instead of passing the knife between the tendon and the skin, and cutting toward the bone. A small piece of plaster laid over the minute incision, is all the dressing that is necessary.

It is common to describe instruments peculiarly constructed for this purpose, but they are unnecessary. Many of the instruments made for tenotomy are too delicate.

Apparatus for extension is immediately applied by some, but in order to secure union of the divided ends of the tendons, by organizing exudations, it may be most safe to postpone this for a few days, and then to make the extension very gradually. It is not known that the tendo achillis, divided sub-cutaneously in early life, in the human subject, has ever failed to unite; but in an experiment which I made, some years ago, upon a dog, the divided tendo achillis united only by shreds of its investing sheath, which indeed may never have been divided.

It is suspected that the uniform success of division of the tendo achillis, as introduced by STROMEYER, gave an unmerited estimate of the importance and utility of the division of tendons and muscles in general. A reaction in this estimate has lead many to discontinue the practice of dividing tendons, except in rare cases of remarkable obstinacy, while others seem still to believe in tenotomy with undiminished zeal.

Among the former is Mr. RICHARD BARWELL, of London, who says, in the preface to his little book, "I studied these maladies from the orthopedic point of view, and while tenotomy was almost a novelty in England; was so charmed with the easy change of form, which, after such an operation, could be produced in most distortions, that I became an almost enthusiastic admirer of the procedure. After, however, following up carefully a large number of these cases, I was pained to find in how many of them the deformity more or less returned, in how many a different, an opposite distortion supervened; while power over the limb was actually injured or destroyed in so large a majority of instances, that its retention appeared absolutely exceptional."

This language sounds very much like that of one temporarily thrown out of balance, by an extreme reaction in opinion, instead of stopping at the safe middle point.

The latest published opinions on the other side, are those of Dr. BAUER, (p. 34 of the little book already referred to), where he says, "The active forms of valgus necessitate the division of the contracted peroneus muscles, or of the whole group of the abductors, as the case may be. This is at least indisputable in inflammation of the tibio tarsal articulation. * * * * * It is difficult to steady the articulation with mechanical appliances in paralysis of the entire motor apparatus of the foot, but it is completely impossible to do so when the malposition of the latter is maintained by retraction of the peronei muscles. We at least have never succeeded by any of the devised mechanical auxiliaries. Meanwhile, the deformity increases and gradually compromises the bones of the tarsus. Between the two evils, we have to choose, and we think that division of the contracted tendons is the lesser."

Now, it is the division of these tendons which, like the peronei run in long ligamentous grooves along the tarsus, which is most objected to. It is claimed that the function of these muscles is often permanently suspended by division, either by not uniting, or by adhering to their sheaths, so as no longer to be able to act upon the bones into which they are normally inserted.

Mr. WM. ADAMS, of London, has been investigating this subject, during the last few years, and has dissected twelve feet, in which tenotomy had been performed. The results of these investigations have been published under the title "On the Reparative Process in Human Tendons." Mr. BARWELL has reduced these results to tabular form, which is here quoted:—

*Table from "Barwell on Club-Foot," ed.* 1863, *analyzed from "Adams on the Reparative Processes in Human Tendons."*

| *No. of Cases.* | *Tendons divided.* | *Results Observed.* | *Time lived after operation.* |
|---|---|---|---|
| I. | Tendo achillis,<br>Tibialis anticus, | Non-union of tibialis anticus............ | 4 days. |
| II. | Tendo achillis,<br>Tibialis anticus,<br>Tibialis posticus,<br>Flexor long. dig. | Non-union of tibialis anticus.<br>" " " flexor longus digitorum. | 11 days. |
| III. left. | Tendo achillis,<br>Tibialis posticus, | Tibialis posticus adhered to the bone. | 23 days. |
| III. right. | Tendo achillis,<br>Tibialis posticus,<br>Tibialis anticus, | Tibialis posticus was supposed to be but was not divided. | 30 days. |
| IV. | Tibialis posticus,...<br>Flexor long. dig.... | Union to all surrounding parts.<br>Non-union, held together by shreds of sheath to which other tendons also adhered. | 18 days. |
| V. | Tendo achillis,<br>Tibialis anticus,<br>Tibialis posticus,<br>Flexor long. dig. | Tibialis posticus and flexor longus digitorum adhered together and to the bone. | 6 weeks. |
| VI. | Tendo achillis,<br>Tibialis anticus,<br>Tibialis posticus,<br>Flexor long. dig. | Tibialis anticus and flexor longus digitorum adhered together and to the bone—ends of tibialis anticus hung together by shreds of sheath. | 6 weeks. |
| In the five next cases, in Mr. Adams' work, the tendo achillis only was divided. | | | |
| VII. | Tendo achillis,<br>Tibialis posticus,<br>Flexor long. dig. | Non-union of tibialis anticus,<br>Neither retraction nor extension of the flexor longus digitorum. | Several years. |

### *Analysis of the Preceding Table.*

Division of the Tendo Achillis, 12 Cases.

United, in Cases 12 Not United, in Cases 00

Division of the Tibialis Anticus, 4 Cases.

United, in Cases 1 Not United, in Cases 3

Adherent to surrounding parts, equally destroying the function of the muscle, in Case 1

Division of the Tibialis Posticus, 7 Cases.

Not Divided, in Case 1

United, in Cases 3 Not United, in Cases 3

Adherent to bone or surrounding parts, suspending the function of the muscles, in Cases 3, that is in all cases of non-union.

Division of the Flexor Longus Digitorum, 5 Cases.

Union in Case 1 Non-Union, in Cases 4

Adherent to surrounding parts, (among the cases classed non-union), in Cases, 2

From this analysis we may well hesitate before dividing any tendon about the foot, except the tendo achillis. If the result in these cases is of any value, the division of these tendons should only be practiced in instances in which, from permanent loss, or paralysis of the opposing muscles; a permanent loss of muscular contraction is desirable in the muscles whose tendons are to be divided.

The following interesting observations and experiments, by Dr. L. T. HEWINS, of Loda, Iroquois County, Illinois, show the influence of young age, upon the activity of cicatrix formation, to connect the divided ends of tendons, or to pull them together.

Upon a dog four years old he failed. Upon dogs ten days old, and three months old, he succeeded after removing portions of tendons. He also succeeded perfectly upon a rabbit. He observed the reproduction of tendon, or substitute for it, in the extensor digitorum manus in one man 35 years old, three-fourths of an inch, having sloughed off, and in another man aged 38, half an inch, having been lost by sloughing.

These latter cases were successes under difficulties, the wounds being open and granulating, and presenting the conditions most favoring the agglutination of the tendons to the bones and other surrounding parts. The influence of motion in elongating adhesions, and reducing shapeless masses of newly organized material to the shape and function of tendon, whether permanent or temporary, by its gradual shortening and disappearance, is well illustrated.

*Loda, Ill., Sept.* 12, 1862.

Divided the tendon of a healthy dog, about four years old, corresponding to the tendo achillis in man. Removed a section of the tendon so as to be sure if I could get reproduction of tendon in an animal of that age. Dressed the limb with splints and rollers, to prevent motion.

Sept. 20th, removed dressing from the limb; external wound healing kindly; no evidence of growth of tendon.

Oct. 2d, examined limb; no evidence of reproductive fascia, both superficial and deep-seated, are quite adherent to the divided ends of the tendon.

Oct. 15th, removed dressing from limb; no elongation of tendon; fascia and tendon uniting; fascia more firm than at former examination, and evidently thickening.

Dec. 1st, examined the divided tendon; find no evidence of growth in length of tendon. Fascia have united with the divided ends of the tendon to form a connecting link between the divided parts. The dog walks with a hobbling gait.

Sept. 12, 1863, one year after the division of the tendon in the above case; there is no evidence of reproduction of tendon. The divided ends of the tendon may be felt through the integument and fascia very firm; dog has a hobbling gait; is permanently lame.

Sept. 13, 1863, divided the tendon in a dog about 10 days old, corresponding to the one divided in the former case, and a portion of the tendon removed; dressed the limb to keep it at rest; dog seemed entirely healthy.

Sept. 20th, dressed the limb of the dog having the divided tendon; there is evident prolongation of tendon.

Oct. 2d, dressed young dog's leg; tendon manifestly extending so as nearly to unite.

Oct. 12th, tendon not yet united; kept on the dressing as before.

Oct. 23d, tendon not completely united, but divided ends approaching each other.

Nov. 15th, examined the young dog's leg; found the tendon entirely united; having a good degree of firmness; dog walks without halting.

Dec. 25th, divided tendon seems as strong as the undivided one of the other leg; dog walks without limping.

Feb. 2, 1864, divided tendon of a dog three months old; dressed, after removing a portion of tendon, so as to keep from motion.

Feb. 10th, dressed the young dog's leg; wound in integument healing kindly; evident formation of new tendon.

Feb. 20th, dressed the limb; tendon still growing in length.

March 2d, dressed leg; found divided ends of tendon approaching each other.

April 1st, tendon fully formed and pretty firmly united; wound has healed kindly; dog walks well.

March 3, 1864, divided the tendon in the leg of a rabbit; kept the animal still; dressed the limb to keep motionless.

March 10th, dressed the wound; looks well; tendonous organization evidently going on well.

March 20th, tendon elongated; union hopeful.

March 30th, tendon fully formed, but soft.

April 15th, tendon fully formed and more firm; animal walks well. This young animal seemed very healthy.

Nov. 4, 1862, D. S., (a German by birth), a healthy man, aged 35 years, had the extensor tendon of the middle finger, on the left hand, divided by a corn knife; wound was neglected about 14 days, by which time tendon had ulcerated, and about three-fourths of an inch of the entire tendon had sloughed out, when he applied to me for treatment; dressed the hand and kept finger extended and at rest; attempted to subdue inflammation in hand as soon as possible, (which was at the time extensive), and arrest ulceration of tendon and its necessary destruction.

After 12 weeks, new tendon had been produced to supply the waste made by previous ulceration, and the finger restored to its normal action.

April 15, 1864, Mr. J. D., a man aged 38 years, had his index finger, on left hand, seriously injured by contused wound on hand-car. April 25th, applied to me for treatment; I found about one-half inch of the extensor tendon of the finger sloughed off. I have dressed and watched the finger carefully to this date, June 2d, 1864, and by this time new tendon has formed, but is soft—I think we shall have good finger.

Mons. BOUVIER is quoted by BARWELL as having divided, in 1842, in a dog, the flexor carpi radialis, the flexor carpi ulnaris, the flexor digitorum sublimis, and the flexor digitorum profundus. In none of these did the sub-cutaneous wound unite so as to restore the use of the parts. In another experiment, the tendons did not unite at all; in another, the severed structures were massed together. Mons. BOULEY met with the last result in an experiment upon a horse.

It is probable that in some of these cases of massing together, there would be afterward an absorbtion of portions of organized exudations, which impede the movements of tendons, like that which occurs after a general union of tissues in the neighborhood of fractures, so that the result finally would not be quite as bad as might be inferred from these statements.

An objection strongly urged, even to the division of the tendo achillis, is that the "cicatrix contraction" which attends all solutions of continuity, united by the interposition of extensive organized exudations, gradually diminishes the distance between the cut extremities of the divided tendon, so that they are finally brought nearly or quite together. This makes a bad compensation for the advantage gained at first, by the necessity of the wearing of apparatus to prevent the recurrence of the deformity, while this process of cicatrix contraction is going on. In the treatment without tenotomy, the muscles is from the first made to grow longer, by a change of its nutrition induced by the force gradually and persistently applied, rendering the progress at first more slow, while in the treatment by tenotomy, this growing of the muscle to a greater length has afterward to be secured, when the case fallaciously seems to have been completed, and perhaps after the case has passed from under the supervision of the surgeon.

The following is BARWELL'S language upon this subject:—

"The reunion of the tendo achillis, after its division for talipes equinus, is almost a certainty, but it" (the division) "permanently weakens the muscles, nor is such a procedure, as a rule, an efficient cure of the disease; partly because the gastrocnemeus and soleus are not the principal muscles affected, and generally have very little to do with the malposture; partly, because contraction is sure to recur." (p. 120.)

Notwithstanding all this, however, there are occasional intances in which even Mr. BARWELL, anti tenotomist as he is, would divide the tendo achillis.

"I do not mean to deny that, occasionally, when there is either great want of development, or great degeneration, it may be necessary to divide the tendo achillis, but it should always be avoided if possible, since it is merely a temporary expedient, which always leaves behind it a certain deformity." (p. 127.)

In contrast with this again is the language of BAUER. (p. 24.)

"As a general thing, you have only to deal with the contracted muscles, and *division* is the sovereign remedy. But if the case has existed from infancy, the bones have in form accommodated themselves to their abnormal position; the tibio tarsal articulation is crippled; then the prognosis is rendered doubtful, and the case may be irremediable."

"It is a common observation of orthopedic surgeons, that the relief of contracted muscles by tenotomy reacts most favorably upon the nutrition of the affected extremity, and nutritive supply promotes, self-evidently, its growth and development. Passive motion coöperates in the same direction."

A question of interest here arises as to what part the division of the tendo achillis takes in the restoration of the muscular function.

If the congestion of the muscle occasioned by the increased supply of blood to the tendon beyond, for the repair of its wound, favors a better nutrition, and consequent restoration of nervous power, it might be supposed that a seton or an issue applied nearer to the muscle to be affected, would, from the proximity of inflammation, do better by exciting more action in the muscle.

It is more probable that the movements of flexion and extension, which attend the treatment following the division of the tendon, and subsequent to the reunion of the divided tendon, gradually induces a lengthening of the muscular fibrils, and this lengthening is a necessary condition to their shortening under the irritation of electricity or any other irritant.

This opposing force should be either elastic or alternating, in order to obtain the most stimulating effect upon the muscles in process of restoration; permitting frequent exercise of contraction, with yielding force so graduated as to restore the length of the muscles upon the decline or cessation of their contraction.

The alternating movements of the tendons of the still paralyzed antagonist muscles, first pushing and then pulling these tendons, and in a minor degree pushing and pulling the muscles themselves, invites a flow of blood to the muscular substance, favoring its continued healthy nutrition, and the earliest possible revival of nervous power, when the paralyzing cause, residing in the brain, in the spinal cord, or in the course of the nerves, whether from organic lesion or sympathetic action, is removed.

If the cause of the paralysis is such a destruction of nervous substance as to result in complete and permanent paralysis, the alternating movements of the muscles will at least tend to preserve their volume, by keeping up their nutrition, by making it mechanically possible for the blood to circulate through all their cappillaries; motion being as essential to the freest circulation through the muscles as through the lungs.

The general health then has the benefit of a well distributed circulation, in addition to the local advantages of attention to this indication.

This plan of yielding force, called by Dr. Henry G. Davis, "elastic extension," is very properly denominated by him, the "American Plan," and to him is due the merit of having been the first to employ it systematically, and with a full appreciation of its value; acting in a manner similar to that of muscles, alternating in the extent of their movements with the alternations of the degrees of resistance to be overcome.

Apparently from ignorance of American medical literature, Barwell claims this plan as his own. This is one of the instances in which several claimants for originality, my be equally honest and original, the merit, however, consisting in the application of some other invention, which makes a revolution of the given art, not only easy but unavoidable.

In this case, the invention at the bottom, is the manufacture of elastic rubber, placing in every one's hands a most facile means of meeting an indication which the older surgeons saw, but had no ready means of accomplishing. (See Trans. Am. Med. Assoc., 1863.)

In cases affording obstinate resistance to reduction by extension, the progress can be greatly facilitated by the occasional application of force, while the patient is insensible from the influence of ether.

The same condition is artificially produced which occurs in a subluxation or sprain. The most tense ligamentous fibres are torn without a complete rupture. The investments of the muscular fibres in the shortened muscle are either slightly torn interstitially, or put upon extreme tension. All this is followed by increased vascularity, which is favorable to change of tissue, in obedience to the tension afterward applied to it for the purpose of elongation.

This has been a common practice among American surgeons for many years, though BARWELL, strangely enough, claims it as his peculiar invention. He says, with much apparent satisfaction, (p. 116,) "This is also a procedure of *my own* adaptation to these diseases, and is one from which very great advantage may be drawn." He very properly goes on to say, "I would limit its employment to severe cases, and would caution surgeons against the use of violence; since, when once the muscular power is annihilated by the anesthetic, very little force is required to place the foot in a normal position."

*Electricity.*

Electricity has been employed to remove the condition of the muscles upon which the deformity has been supposed to depend.

This subject cannot better be illustrated than by quoting from representative writers, who take opposite positions.

BAUER, already so often quoted, more for the recent date of his publication than for its scientific value, says:—

"The most efficacious remedy in behalf of innervation is electricity. It should be used with assiduity every day, and for months in continuation. It will stimlate the existing mobility, and prevent structural decay." * * * * "Electricity is the substitute for volition, and the best local gymnastic agent." "Next are friction with alcoholic liquids, with phosphorated oil, (phosphorus 3 grains dissolved in an ounce of warm almond oil), with the flesh-brush, with or without cold irrigation."

We are left to infer that he would apply the electric current to the contracted muscles, with the intention of relieving the spasm upon which the contraction is supposed to depend. This question of spasm has been already sufficiently discussed, and it may be proper to add, that as a curative agent, the galvanic current should not be applied to the muscles whose tendons it has been found necessary to divide, but to the elongated muscles, whose partial or total paralysis has permitted the shortening of their antagonist muscles.

It is obvious that when, by unresisted tonic contraction, the muscular fibres and their fasciæ have shortened to their utmost, neither electricity nor the prick of a pin can make them shorten any more. A galvanic current can make no impression which is known by movements, because this agent and other irritants only produce contraction. If, however, the muscular fibrils and their investments are first made to grow longer, by frequently repeated pulls upon them, or by constant force varying in intensity, thus restoring the muscle to a greater or less extent, to the possibility of performing its natural function: that of producing *motion*, instead of the one to which it had degenerated, that of holding parts in *position*, is the function of ligaments; then, after so much progress has been made towards the cure, it might be expected that electricity would index it by the contractions which would result from its application.

It is difficult to see, however, on what rational principle electricity should be applied to the shortened muscles with any other intention than to determine whether they could shorten any more, or to ascertain, in the progress of treatment, in a case in which a muscle had been shortened, and degenerated beyond the possibility of exciting contractions by the electric current, whether any progress had been made, or, perhaps, to throw light upon the probable replacement of the muscular substance by fatty degeneration. In the latter case, electricity could not produce movement.

The notion of Bauer, that we have only to deal with the "contracted muscles," is certainly in forgetfulness of all correct pathology. He details, in his book, cases of paralysis of the inferior extremity, followed by permanent extension of the foot, beginning with painless contraction of the extensor muscles.

Now, what would electricity do with these muscles? It might make them contract more disproportionately, or if in too strong currents it might exhaust their excitability. What would tenotomy do to them? It would permit a greater degree of shortening of the muscle affected than could otherwise take place. We have something else to deal with than the contracted muscles.

In these cases of paralysis of all the muscles of the leg, there was an attempt at restoration of muscular power, commencing in the triceps extensor pedis. The restored contraction of these muscles having no resistence to oppose, followed the usual law of shortening, and of acquiring a more limited space of contraction, or from utter want of pull upon them, a fixedness in the shortest space, to be followed by fatty degeneration, or by absorption of the proper substance of the muscles, and a condition of inelasticity in the muscular investments. In all such cases the early use of power to counteract the muscular contraction is an imperative indication; partly to obviate the permanent contraction of the muscles which are in the process of restoration of their proper function; and partly to give time for the restoration cf contractibility in the paralyzed antagonising muscles which are slower in the process of restoration.

If in the restoration of muscular contraction, referred to in these cases of paraplegia, both sets of muscles had been supplied alike, by nervous power, no deformity would have resulted. There remained a relative paralysis of the flexors of the foot—the tibialis anticus, peroneus tertius, and long extensors of the toes. If this is so, the electric current should be applied to the latter muscles, rather than to the calf of the leg.

The following quotation from R. B. TODD'S "Clinical Lectures on Paralysis and Diseases of the Nervous System," Lindsay & Blakiston's ed. p. 152, will here be in point.

"You will often be consulted as to some expedient for promoting the restoration of paralyzed limbs to their normal condition. To this question, after having given a fair trial to the various means which have been proposed for this purpose, I must reply that I know of nothing which more decidedly benefits paralyzed limbs than a regular system of exercise; active when the patient is capable of it, passive if otherwise.

"As to the use of electricity, which is now much in vogue, or strychnia, which has been recommended, I feel satisfied, as the result of a large experience, that the former requires to be used with much caution, and that the latter is apt to do mischief, and never does good. I have seen cases in which, after the employment of electricity for some time, that agent has apparently brought on pain in the head, and has excited something like an inflammatory process in the brain. And so strychnia will also induce an analogous condition of the brain, *and will increase the rigidity* of the paralyzed muscles. Some good may occasionally be effected by the use of friction or cold water, or shampooing, all of which tend to improve the general nutrition of the nerves and muscles."

*Apparatus.*

In the older plans of treatment, still retained by many of our surgeons of reputation, some immovable and inelastic frame of wood or iron, properly padded, was employed to bring the foot around into proper position; the apparatus being changed for another of different shape as the restoration progressed, or adapted with joints to change with the changing shape of the foot.

The simplest and oldest form is a flat splint, to apply to the leg, with a flat, thin foot-piece, the edge of which was fastened upon the end of the splint, in the form of a cross, upon which the foot and leg was bound by roller-bandages. In contrast with the simplicity of this, are the complicated machines, invented by SCARPA, SCONTETTEN, and others, in the beginning of the great awakening upon the subject of orthopedia, about thirty years ago.

SCARPA'S shoe has an iron sole, an iron heel-piece at right angles with this, and a brace running up the leg, while a spring attached to the side of the shoe, gives a pull with some elasticity for straightening the incurved foot; all this is properly padded and provided with straps and buckles. The vertical brace passes up on the projecting or convex side—upon the outer side in talipes varus. The illustration, fig. 5, shows the iron frame-work of this complicated machine.

*Explanation.*

The shoe is in a straight position, *a* the sole, *b* the semicircular portion to embrace the heel, a portion behind is cut away, leaving a hole for the end of the heel to protrude; *c* the horizontal spring for abduction of the foot; *e* a hinge in the upright portion; *f* a triangular screw-head which is turned with a key, and causes the point of the instrument to turn down; *g* another hinge; *h* another triangular screw-head, which, being turned with a key, bends the foot part outward; *i* the upright shaft or brace; *k* the semicircular part to go round the leg, and act as a fixed point of the apparatus.

Fig. 5.

Fig. 6.

SCONTETTEN'S apparatus differs from SCARPA'S chiefly in having two shafts, one passing up on each side of the leg. Fig. 6 illustrate it without all its padding.

Dr. BAUER, in his work already so often quoted, employs a slight modification of SCONTETTEN'S apparatus as the utmost advance in the art at the present time.

These machines, however, are not well adapted to any species but T.

equinus and T. varus, and for each varying size of foot, an expensive apparatus must be made. They are uncomfortable, extremely liable to produce ulceration; almost destitute of elasticity, acting chiefly upon the ankle-joint, and moving the foot as a whole, failing to move the tarsal joints upon each other as is done when the foot is grasped by the hand. They are difficult to make except by skilled instrument makers. The desideratum is a method which is within the skill of any person of ordinary ingenuity, to be made of materials always at hand and free from expensiveness.

The use of adhesive plaster, introduced about the year 1850, was a great advance in the art. The method consists in cutting strips of convenient width and long enough to envelop the foot and pass up the leg nearly to the knee, there to be fastened in place by circular strips passing round the leg, over which the upright strip (or strips, for there must usually be several of them), are turned so as to clinch them to prevent their sliding.

For T. varus the plaster ascends on the outside, and for T. plantaris, and T. valgus on the inside, and for simple T. equinus, on both sides. It is sometimes found convenient to carry the fastening above the knee for greater space for application of the plaster.

This expedient holds the foot in the position in which it is placed by the hand of the surgeon, except a little sliding that plaster will always be guilty of. It very soon occurred to me that a piece of elastic rubber ribbon could be interposed in the vertical strip of adhesive plaster, so as not simply to hold the foot in the position in which it was left by the hand, but to be constantly gaining by a yielding but unintermitting stretch night and day, gradually elongating the opposing muscles and ligaments, and by the slight mobility attending the elastic rubber, permitting some passive motion in the muscles assisted by the elastic appliance, whereby their circulation is increased, with a more rapid nutrition and a more speedy accommodation to their altered length of contraction.

I for sometime supposed this to be the last advance of which the art was capable, but, ulceration sometimes occurred upon the edge of the foot, where the circulation was too much impeded by the circular compression of the plaster around the foot. There seemed to be a lack of some expedient by which the fold of the tarsus could be straightened out, so as to restore the foot to its normal breadth. An obstinate case, attended with ulceration of a delicate skin, led me to devise an appliance which is a tolerable substitute for the hand; but before describing it, a few pages must be devoted to the plan of treatment pursued by Mr. BARWELL, to explain which, his book (on Club-Foot, &c.) seems to have been chiefly written.

The peculiarity of BARWELL'S plan consists in his method of attaching the proximal end of his tension apparatus, which is this:—Starting with the idea of making the artificial tension the exact complement of that of the partially paralyzed muscles; he aims to act as nearly as possible upon the same bones to which these muscles are attached, (and in the same direction), by adhesive plaster fastenings, while the points from which the pull comes are the origins of these muscles.

Thus, for T. varus, the fastening is made on the exterior anterior side of the upper part of the leg, at a point over the origins of the peronei muscles, in such a way as to get two-thirds of the length of the leg for the position of the rubber spring upon which he relies for the pull.

The lower attachment is made to imitate as nearly as posible the insertions of these muscles; but for retention to the skin, the lower adhesive plaster passing downward over the cuboid and fifth metatarsal bones must cross the bottom of the foot, and fasten upon the inner side above the sole. In order to get a retention of the rubber spring upon the upper part of the leg, a broad strip of adhesive plaster, twice the length of the leg, is applied over the course of the peronei muscles, over the fibula, and upon this, a piece of tin, a little narrower than the plaster, is laid, and the lower end of the plaster turned up over it, so that the inside (or sticky side) is outside, for adhering to the roller that applies round the whole, to hold it fast. The upper end of the tin is turned over from the leg, and has a hole punched in it, and into this hole an eyelet is inserted; a similar eyelet is inserted in the adhesive plaster which passes across the bottom of the foot, and between these is stretched a rubber spring. By the combination of two or more of these expedients, he is enabled to obtain tension which imitates the combined action of the peroneus longus and p. brevis, passing behind the external malleolus, and the peroneus tertius, passing in front.

For talipes valgus, he makes a similar appliance on the inner side of the leg and foot, to supply the deficiency of the partially paralyzed tibialis anticus and tibialis posticus. The pull must here be in two directions as in the other case.

In talipes plantaris, (flat-foot), he makes a direct lift upon the hollow of the foot, by an anterior appliance compensating the deficient lift of the tibialis anticus.

In talipes equino dorsalis, he makes also a direct lift further forward. He explains this deformity as being the direct opposite of talipes plantaris or flat-foot, in which the medio-tarsal joint sinks too low, hence it must be lifted up; while in talipes equino dorsalis, the same joint rises too high, while by the contraction of the tibialis posticus, the peroneus longus, the p. brevis, and the flexor longus digitorum, the metatarsus is flexed or drawn down, bringing the toes to the ground, while again the instep or "waist" of the foot rises too high. He thinks the action of the sural muscles, through the tendo achillis, upon the calcaneum, a minor element in the deformity, and hence a particular objection to the division of the tendo achillis, in addition to the general objection arising from permanent injury to the tendon.

The account would be more nearly correct to say, that in addition to the contraction of the tibialis posticus and flexor longus digitorum, the foot is arched too high by the shortened condition of the adductor pollicis, the flexor brevis digitorum perforans, the abductor minimi digiti, and the musculus accessorius, with shortening of the plantor fascia to correspond with this disproportionate contraction of these muscles.

The pull directly in the line of these tendons, besides being a refinement of treatment difficult, and sometimes impossible to execute, is one which acts at a great mechanical disadvantage, implying a greater pressure upon the skin, to accomplish a given amount of change of position, than would be required by a direct pull.

If it had been the design of nature to make only slow movements of the extremities, there would have been nothing gained by binding down the tendons under transverse ligamentous substances as they pass the joints. A much smaller force would have accomplished the purpose, by acting in a straight line between the origin and the insertion of any muscle. The facility of movement and grace of form secured, by giving the tendons oblique attachments, are elements unnecessary to be regarded by the orthopedist. There is this great disadvantage in this attempt to imitate the oblique action of the muscles: that the pressure upon the skin is three or four times what it is necessary to make it, when the most direct pull is obtained. The importance of gaining the most power with the least pressure upon the skin of the foot can hardly be exaggerated. Ulceration of the foot, where the pressure applies, is the greatest difficulty which it has been the study of surgeons to avoid.

It cannot be said that the muscle which is partially paralyzed is more assisted by the oblique pull than by the direct, for the passive motion of the muscle is communicated by the push and pull of the tendon; and this *to* and *fro* movement, must be the same for a given amount of motion of the parts to which the tendon is attached, whether the movement is effected by an oblique pull in the direction of the attached end of the tendon, or by a power acting at a less mechanical disadvantage, like the hand of the operator, or any apparatus which acts in a similar manner.

*Illustrations of Barwell's Method.*

Fig. 7 shows the manner of applying the plaster over the tibia, and the tin over it, and the plaster under the sole of the foot for T. plantaris: *a* a trapezoid piece of plaster into which an eyelet has been fixed, adhering to the sole of the foot, to act as the insertion of the tibialis anticus tendon; *d* a strip of plaster adhering over the tibialis anticus muscle, and having its lower end hanging down more than the length of the limb.

Fig. 7.

Fig. 8.

The letter *d* is upon the upper portion of this free part; *c* a piece of tin carrying at the top a wire loop; *f* the free end of the plaster is turned up on the tin, and a roller applied to hold all fast.

Figure 8 shows the process completed. The lower end of the long piece of plaster has been turned up over the lower end of the tin, and in the figure circular investments of plaster are shown instead of a roller; *g* strip of plaster surrounding the foot, but leaving out the end of the plaster; *b* having an eyelet in it; *l* a rubber spring running from this eyelet in the plaster, which comes from under the sole of the foot, up the leg to the wire loop at the upper end of the tin.

Figure 9 shows the application of the same plan in the treatment of T. varus. Two springs are shown, imitating the action of the peroneus tertius in front of the external malleolus and

Fig. 8.

the peroneus longus, and p. brevis behind the malleolus. *m* A trapezoid piece of plaster applied across the bottom of the foot and having an eyelet. The course of this, under the circular strips, is marked by dotted lines *n*. It is represented as being split so as to embrace the fifth metatarsal bone. *n* The eyelet for the attachment of the rubber spring by a piece of catgut or other strong cord. *o* Circular strapping, covering but one piece of tin, placed just behind the fibula, with its layer of plaster on either side. *v* The remainder of the longitudinal strip of plaster brought down and adherent to the circular ones. *t* A rubber spring assisting the peroneus tertius. *u* A rubber spring assisting the p. long. and p. brev. At the lower part of the attachment of the spring, marked *u*, is an arrangement for changing the direction of the force, by an attachment around the limb. *v* A short piece of rubber tube covering a hook, by which the spring is attached to the eyelet in the upper end of the tin. All the attachments are covered in the same way in practice to shield the hooks from the clothes.

In obtaining the pull from a space directly over the elongated muscles, by the plaster and tin appliances, a very considerable pressure is produced over the whole circumference of the part. We know that a moderate pressure like that produced in health by the skin and fasciæ, and by a laced stocking, when these are relaxed in varcose veins of the extremities, is favorable to muscular tone, but a greater degree of pressure, like that produced by ligating a member for cramp, is unfavorable to muscular contraction. It is feared that in this method of obtaining the resistance to the pull of the artificial muscle, directly over the muscle whose weakness is to be compensated, there may be a temptation, in hands more unskilful than those of Mr. Barwell, to bind the limb so tightly as to interfere with the most rapid restoration of the muscular function. This tightness is almost necessary, in order to prevent the tin with its underlying adhesive plaster from sliding.

The application of adhesive plaster to the foot, as employed by Barwell, does not materially differ from the method for many years in common use. The plaster cannot be stuck to the skin as the tendon is stuck to the bone. It must have a considerable breadth of attachment or it will slide off. This necessary extent of surface cannot easily be obtained upon the foot without carrying the plaster round upon the opposite edge, so that its pull must approximate the bones of the metatarsus and of the phalanges. This force is the direct opposite of that which is produced upon an inverted club-foot (talipes varus) by walking upon it. The weight of the body, in walking, comes upon the cuboid, the fifth metatarsal bone, and corresponding phalangeal bone until, by folding and twisting, the foot is still further turned, so as to bring the weight of the body upon its dorsum.

The plaster takes hold of the opposite or inner border, (in talipes varus), and passing under the foot and up on the outside pulls in the opposite direction. In all this there is no tendency to take the longitudinal fold or doubling out of the foot. The force simply untwists the malposition of the cuboid in relation to the calcaneum, and the cuneiform bones in relation to the scaphoid, and, more than all the others, the scaphoid in relation to the astragalus. To the extent of the tilting of the astragalus in the ankle-joint, and the sliding of the calcaneum upon the astragalus, these deviations are also corrected.

It is obvious, by a glance at the skeleton, that an important agency in reducing the slight dislocation of the cuniform bones upon the scaphoid, and the principal dislocation of the scaphoid upon the astragalus, is the unfolding of the foot to give it transverse breadth. This is one of the most important indications in cases in which the patients have been some time walking. It is easy enough to answer this indication with the thumb and fingers taking hold of the foot and twisting it in directions opposite to those of the distortion; but the thumb and fingers soon tire out. It is possible to employ a succession of hands for that purpose, and this would probably be as effectual as any more artificial method. The desideratum is the invention of apparatus which will do what the thumb and fingers can do, and to do it without tiring out, and without danger of producing ulceration from the persistency of unyielding pressure. The device to answer this end, without much expense, and in a method so easy of execution that it can be readjusted every day or two, is simply thus:—

For a patient 10 years old, take a sheet of gutta-percha one-third of an inch thick, or a sufficient number of thinner sheets to make that thickness, long enough to encircle the foot, and wide enough to extend from the middle-joint of the phalanges to the medio tarsal articulation, between the scaphoid and astragalus above, and the cuboid and calcaneum below. Apply upon both surfaces of the gutta-percha an investment of muslin of good strength, and lay the whole, thus prepared, into a pan of water nearly boiling hot. While the softening process is going on, the foot should be wrapped with a roller, protecting the prominent points with pledgets of lint or cotton.

As soon as the gutta-percha is thoroughly softened, it is taken out, still lying between its muslin investments, and so applied that its ends come together on the outside of the foot in talipes varus, where the two extremes of gutta-percha should be welded by pressure between the thumb and fingers, previously dipped into cold water to keep the material from sticking to the fingers.

In talipes valgus the extremes of gutta-percha meet and project on the inner or median side of the foot. While the material is yet warm and yielding, a square piece of pasteboard is laid upon the dorsal surface of the foot with a corresponding piece of oiled silk or rubber cloth, underlying it, to prevent its softening by the moisture of the wet muslin investment, and a similar piece of pasteboard is applied directly opposite upon the plantar surface.

A common pair of calipers, with screw fastening, is then applied, so that one leg rests upon the pasteboard upon the dorsal, and the other upon the pasteboard upon the plantar surface. The screw is then turned to secure very firm squeezing between the opposing points. This compression is continued until the gutta-percha has become hard and unyielding, except by its elasticity. After this the calipers are removed.

A hole is then punched through the projecting gutta-percha, along side of the metatarsal bone of the little toe in varus, and of the great toe in valgus. Into this hole a cord is inserted, which is fastened to a rubber ribbon or piece of rubber luta or cylinder, which must again have its attachment above by adhesive bands below the knee, above the knee, or by a padded roll to the pelvis which is thereby encircled. This last is the least troublesome attachment, as it can, at any time, be slipped off and put on again. In the last method a knee-cap is necessary to make the tension cord follow the angle of the limb in walking and sitting. The appliance to the foot should be removed and re-applied every day in hot weather, and every alternate day in cold weather, to avoid excoriation from pressure and retained exhalations.

The pressure, if too long applied to a part, without intermission, favors absorption with ulceration; or, if acting with sufficient force, the death of the compressed parts, resulting in sloughing; while the moisture from the skin, with the ammonia which it contains, favors a softening or solution of the cuticle, thus increasing the natural sensitiveness of the parts to pressure.

Figure 10 illustrates the method of applying the apparatus, in talipes varus, to secure tension upon the pelvis.

1 Rubber spring. 2 Buckle for adjustment. 3 Gutta percha investment of the foot, to the outer side of which the tension apparatus is attached. 4 Projection of the toes beyond the investment and above the application of the upper leg of the calipers, applied upon a piece of pasteboard to secure sufficient distribution of pressure. 5 Calipers showing the screw by which the squeezing of the middle portion of the gutta-percha is produced. 6 Knee-bands. 7 Band to which the tension cord is attached, passing obliquely across to the opposite illium. 8 Band around the pelvis to hold the other band from slipping down.

Fig. 10.

Figure 11 illustrates the same method with an attachment above the knee. It is convenient to have a secondary fastening below the knee which is not shown in the cut.

Fig. 11.

The figures refer to the same parts as in the preceding cut. The calipers are supposed to have been removed, and the apparatus to have been fully adjusted. The whole may be inclosed in a moccasin or slipper, to enable the patient to walk about. If the patient is an infant, a stocking may be drawn over the apparatus.

Figures 12 and 13 are accurate copies of photographs of a case of talipes varus in a boy nine years old before treatment, and at the conclusion of treatment, at the end of three months. The flattening down of the tarsus is more perfect than can often be secured without the vertical compression of the foot in the manner just explained. The foot appears shorter than that of the other side, because in the deformed state it had fallen behind the other in growth, but the treatment has spread the foot out effectually, so that there is no danger of a recurrence of the deformity without a nervous derangement capable of producing it from the first.

Fig. 12. Before Treatment.

Fig. 13. After Treatment.

The following quotation from BARWELL, p. 183, aptly illustrates the effect often produced by a theory in hampering one's natural versatility, and driving him to awkward and difficult expedients. The quotation is in explanation of the difficulty of getting room upon an infant's leg for application of plasters, in a child aged six months:—

"A little more difficulty" (than usual) "had arisen from the greater adduction of the foot; this rendered it difficult to fasten on so small a thing as a child's leg and foot, the plaster representing the peroneus brevis, so that the end to which the catgut was fixed did not come against the eyelet in the tin representing the pulley. This is a difficulty which always occurs in children's cases. I find it best overcome by cutting the plaster, which is to represent the tendon of a Y shape, stretching it in the hand that it may not give way on the limb, turning down one of the ends, leaving it very short, and fastening the eyelet into it, while the other two ends are made to adhere, one on the sole and one on the dorsum of the foot, leaving the inner metatarsal bone uncovered. In these cases, also, in spite of any difficulty in knotting it, the catgut must be tied very short; the spring too must be as short as possible."

In this BARWELL recognized, without mentioning or explaining it, the evil of that folding influence upon the foot in talipes varus, arising from pressure of the plaster upon the first metatarsal bone. To avoid this, he stops his dorsal and plantar plasters short of meeting on the tibial side of the foot.

His practical difficulties are very much increased by his theory of getting his pull from over the partially paralyzed muscles. In talipes varus, involving an elongation or loss of action of the peronei muscles, he must get his traction from over the fibula; and he is confined to the length of that bone, because these muscles have only their origins within this space.

By carrying the attachment above the knee there is found plenty of room for the rubber spring, allowing something for the inevitable sliding of the plaster.

By adopting the gutta-percha appliance to the foot, a firm fixture is secured equal to a hand continuously applied, which not only does not increase the abnormal transverse doubling of the foot, but helps to flatten it out, thereby much facilitating the rotation of the top or tibial margin of the foot inward or downward, and the bottom or fibular margin outward or upward.

The origin of this theory was in a correct appreciation of the philosophy of the subject, and the failure of the most complete success, grew out of too close an imitation of nature, where power is lost to gain rapidity of movement and beauty of form. In the artificial removal of deformities, rapidity is only the desire of a fool, and beauty is out of the question; while it is of the utmost importance to avoid all unnecessary pressure upon the skin to which the appliances are attached. The more direct the pull, in imitation of the hand of the operator, the lighter will be the pressure upon the skin, the less the discomfort to the patient, and the more practicable the employment of as much force as the muscles and ligaments will bear without pain in these parts.

The fundamental idea which is at the foundation of my plan of treating talipes, is the invention and application of apparatus in imitation of the action of the human hand.

Iron shoes and all cumbrous inelastic and expensive machinery are thrown away. The restoration of the proper form of the foot is more likely to be the conclusion of the treatment when the muscles, tendons, and ligaments have been elongated without division, by the slower process of growth from nutrition, than when they have been factitiously elongated by division of tendons, and the interposition of cicatriceal material, material which will gradually contract to complete disappearance. The plan here explained makes it practicable to avoid division of the tendo achillis, in cases in which it might be necessary by the old methods, even by the improved plans of BARWELL.

After the treatment is complete, it is useful to steady the foot by a brace running up the side of the leg, having a joint exactly opposite the centre of motion in the ankle. The lower part is made of soft iron, so that the shape can be easily altered, and it is riveted to the sole of a common shoe by two copper rivets, the heads being placed inside the shoe.

The part above the joint, is a flat spring, conveniently made from a worn out saw. The yielding of this spring permits lateral motion at the ankle-joint, while the joint in the apparatus permits flexion and extension. At the top of the spring brace, which should reach about four-fifths of the distance from the ankle to the knee, a cross piece is fastened, made of thick tin or thin iron, of the length of half the circumference of the leg, which serves, when bent to the shape of the leg, to prevent the brace from sliding backward and forward. Over the whole length of the elastic portion of the brace, above the ankle, a leather investment of the circumference of the leg and brace is adapted, which is supplied with eyelets to lace upon the opposite side. The brace is always placed upon the side from which the deviation proceeds. The pull is, therefore, from the brace, so that there can never be any chafing of the skin against it.

This saves all necessity for cushioning it. The brace is always supporting the ankle-joint, and always yielding as the foot treads upon uneven ground. The figures will make this description more intelligible.

In figure 14 all portions of the metal above the ankle are invested by the leather, but in the cut, they are represented as being on the outside.

Fig. 14.

This apparatus will do very well for weak ankles, but should never be trusted, after treatment for talipes varus, as long as the instep is in the least too high. The foot should first, not only have the twist entirely taken out of it, but if a T. varus it should not be left in the least degree a talipes dorsalis. It is entirely practicable, by the method here described, to convert it into a T. plantaris, but this is neither necessary nor desirable. After this thorough removal of the deformity, the surgeon is not likely to be afterward troubled with the case on account of a tendency to a return of the deviation, unless there should be a return of derangement of innervation, such as originally produced it.

It may be noted in closing, that in young infants, previous to walking, and before the infolding of the transverse diameter of the foot from the weight of the body upon its outer margin, the use of the gutta-percha clamp is not very important. The adhesive plaster investment is usually sufficient, but the use of the elastic rubber ribbon is indispensable to satisfactory progress. Where the single ribbon is too delicate, its strength can be increased by doubling. It is convenient to a ttach abuckle or hook at each end of the rubber ribbon, and to work the adhesive strips into them from above and below. The facility for adjustment is then complete.

In order to obviate the lateral pressure of the plaster upon the foot, a sole of leather may be first applied under the foot, made a little wider than the sole of the foot, and the strips of plaster wrapped around this and the foot combined, as is practiced by Dr. H. G. Davis, of New York.

It seems to me that any case of talipes, in a patient under 15 years of age, ought to be restored; but a continuance or a repetition of the derangement of innervation, which originally produced the deformity, may tend to reproduce it, requiring the continued use of an elastic aid to the enfeebled muscles, which may be worn inside of a boot, not differing in principle from the appliances already described, though more delicate and less bulky.

It is not supposed that perfection has yet been attained in this art, nor is it wise to be satisfied with the improvements already made, nor to believe that there is as much known about it now as there ever will be. If, however, we could see what improvements are to come next, we should immediately make them. Experience feels out the future, but sees the past with eyes open.

Imperfect as may be our present attainments, in this branch of the great art, every child born with uncomplicated talipes, in this and subsequent decades, has that claim for complete restoration at the hands of the profession in his own vicinage, which the accessibility of the knowledge how to do it affords.

A walking specimen of talipes, born after this time, will be a disgrace to somebody, who should have known better.

## Figures and Tables

**Fig. 1. f1:**
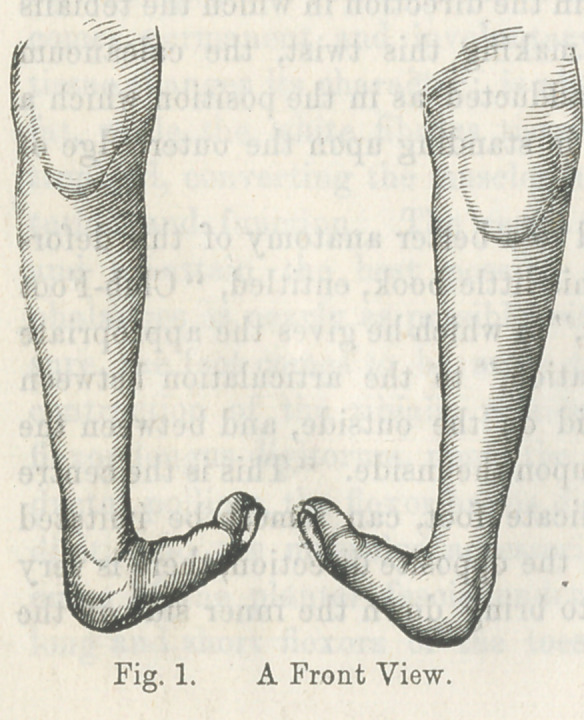


**Fig. 2. f2:**
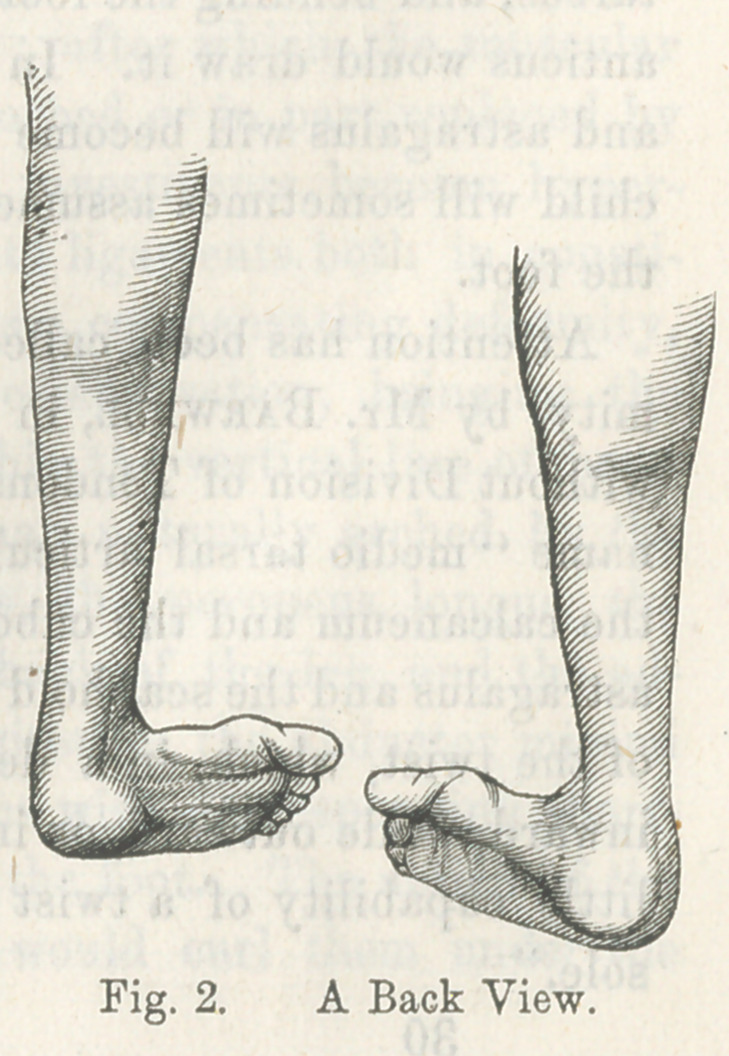


**Fig. 3. f3:**
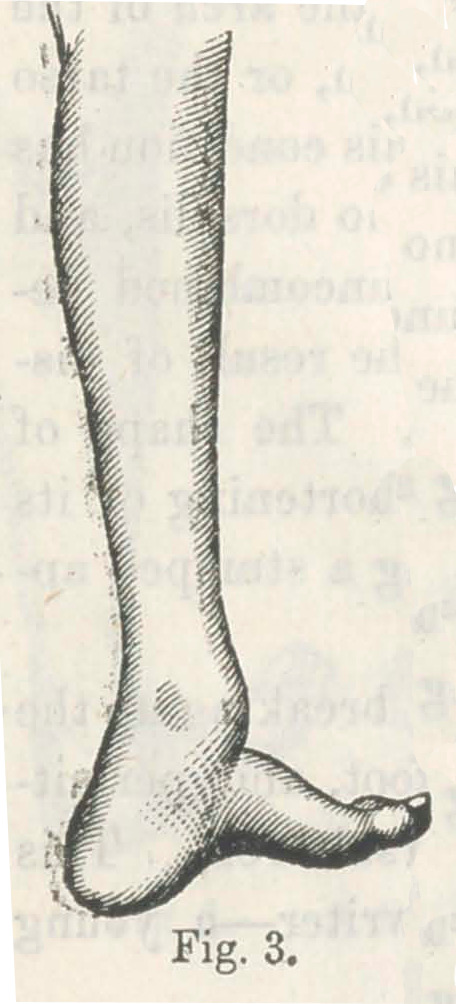


**Fig. 4. f4:**
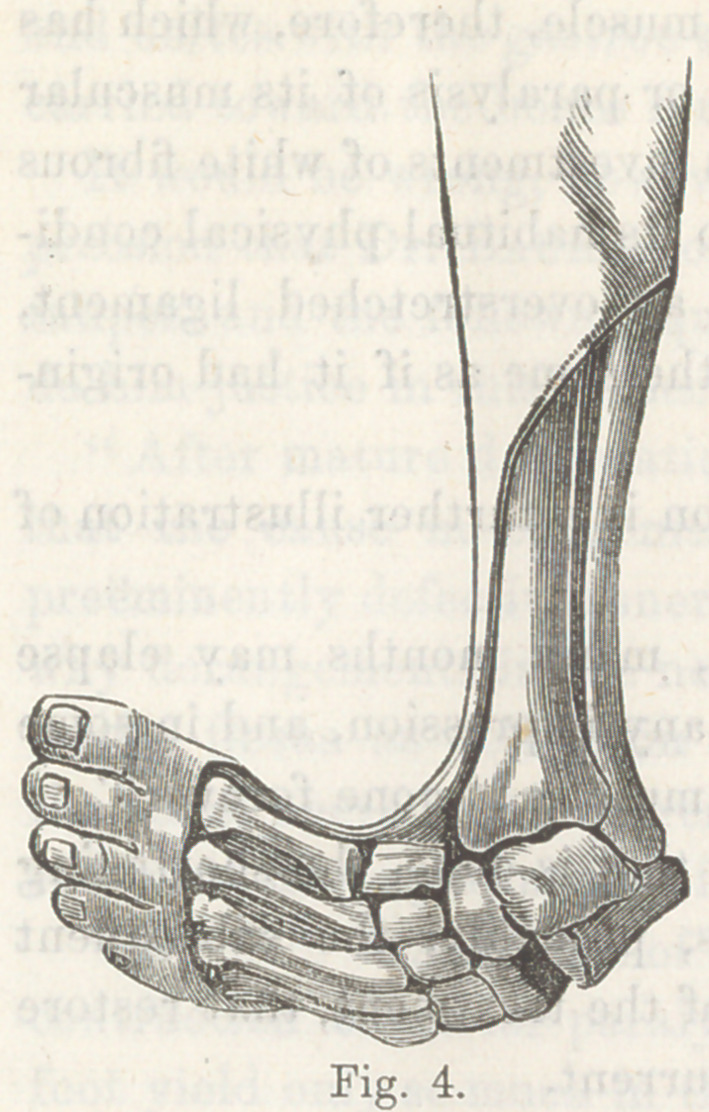


**Fig. 5. f5:**
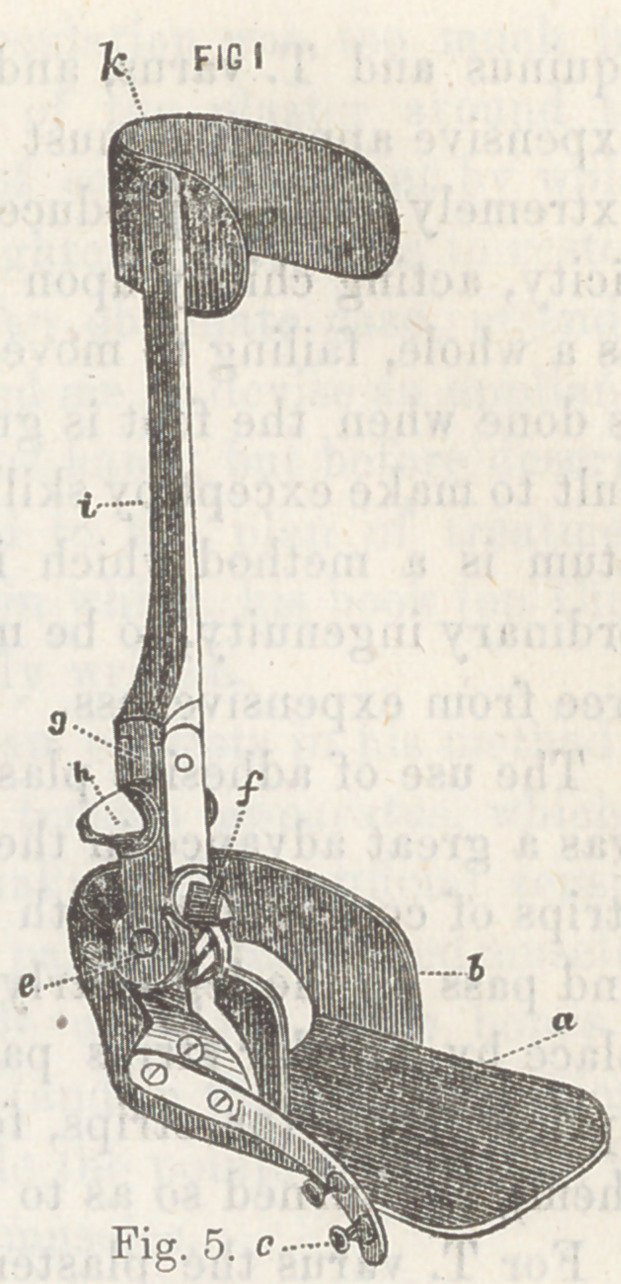


**Fig. 6. f6:**
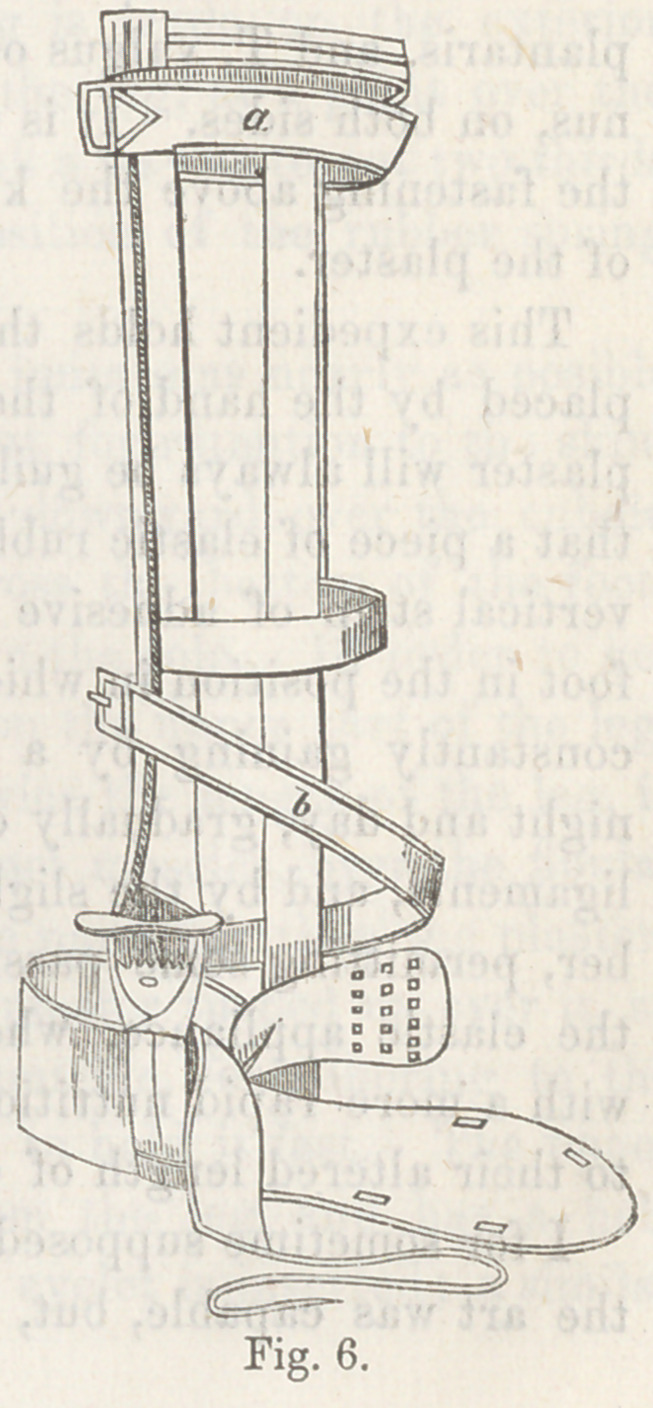


**Fig. 7. f7:**
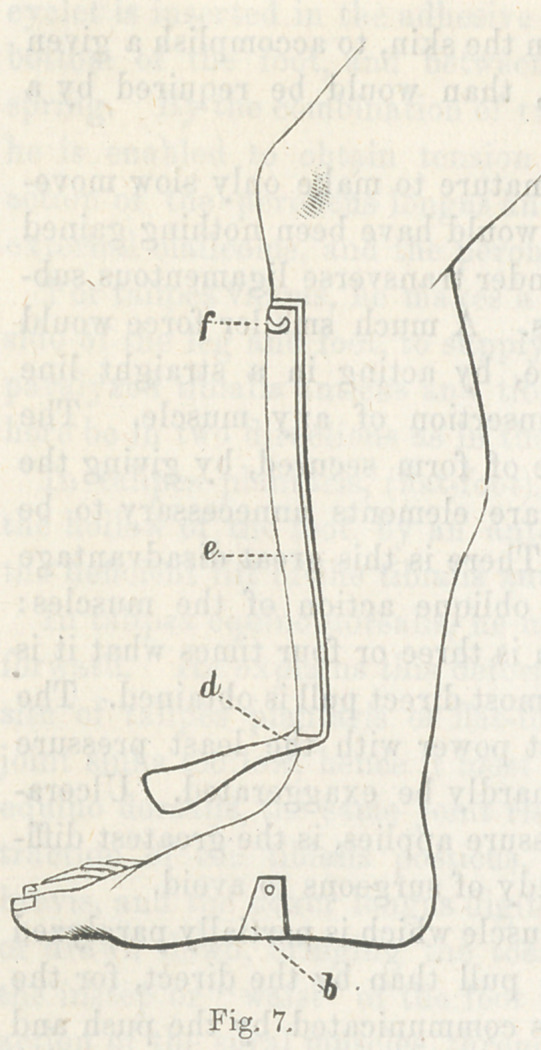


**Fig. 8. f8:**
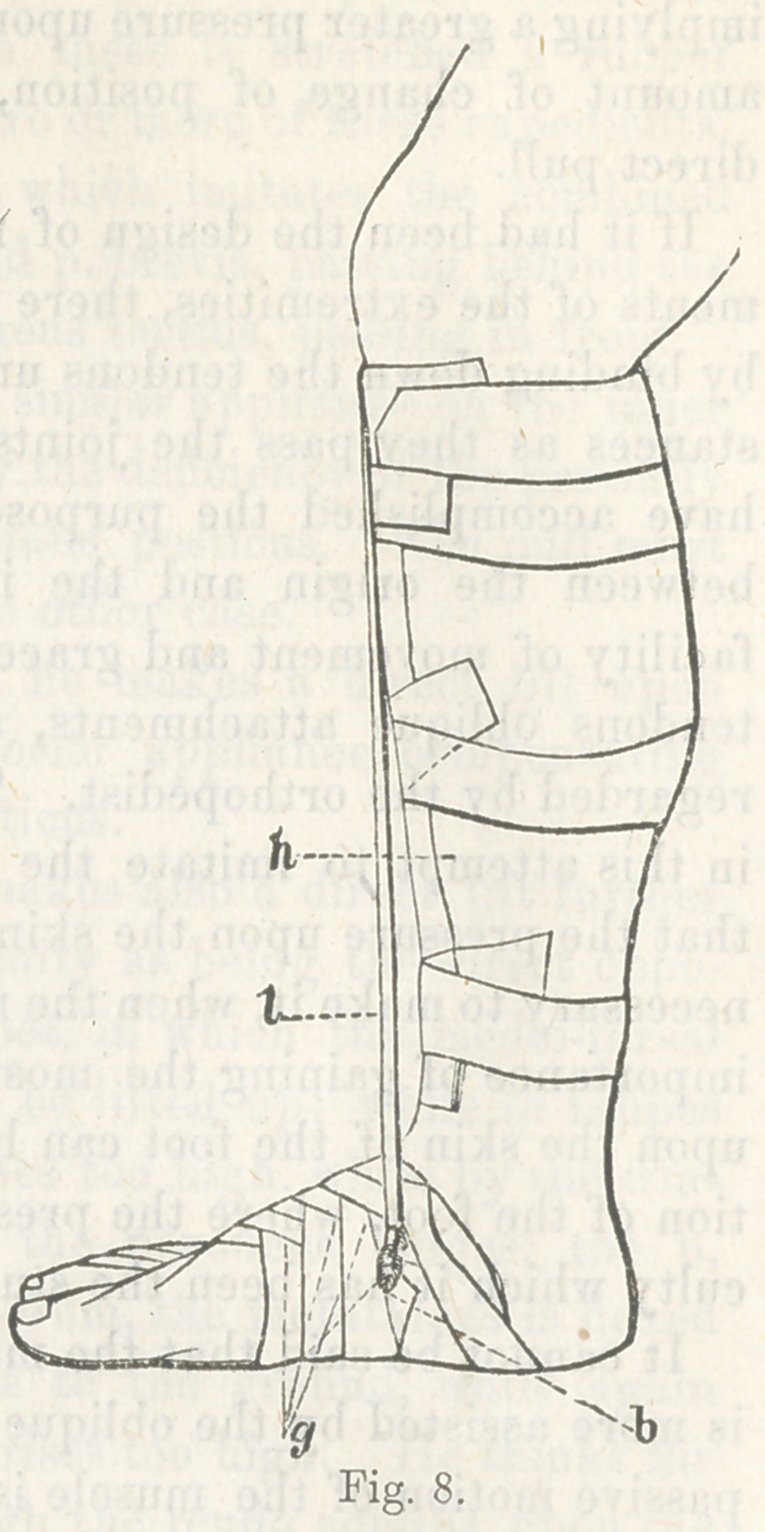


**Fig. 8. f9:**
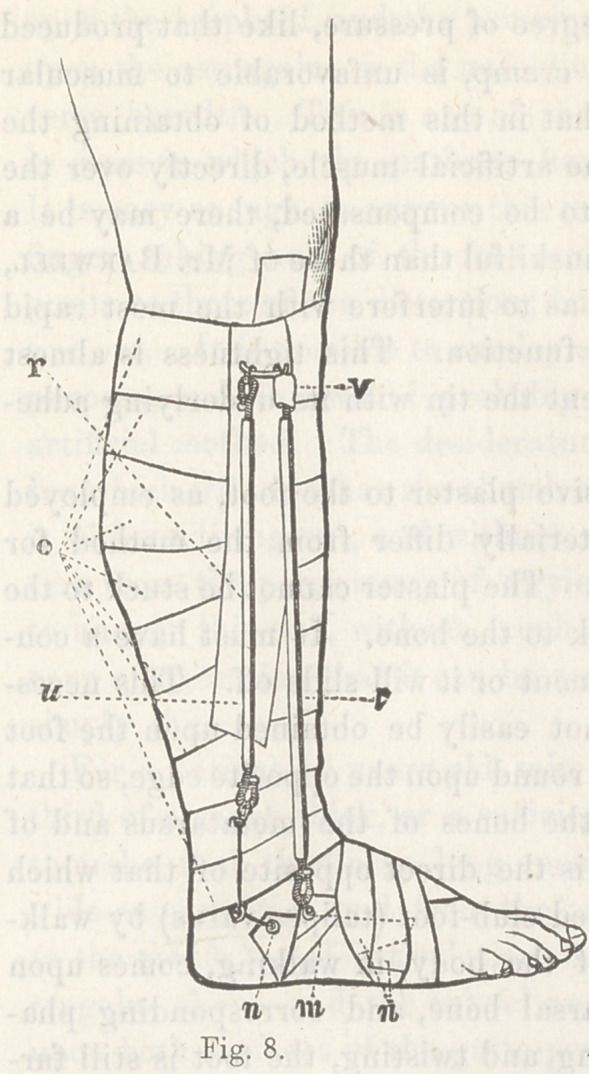


**Fig. 10. f10:**
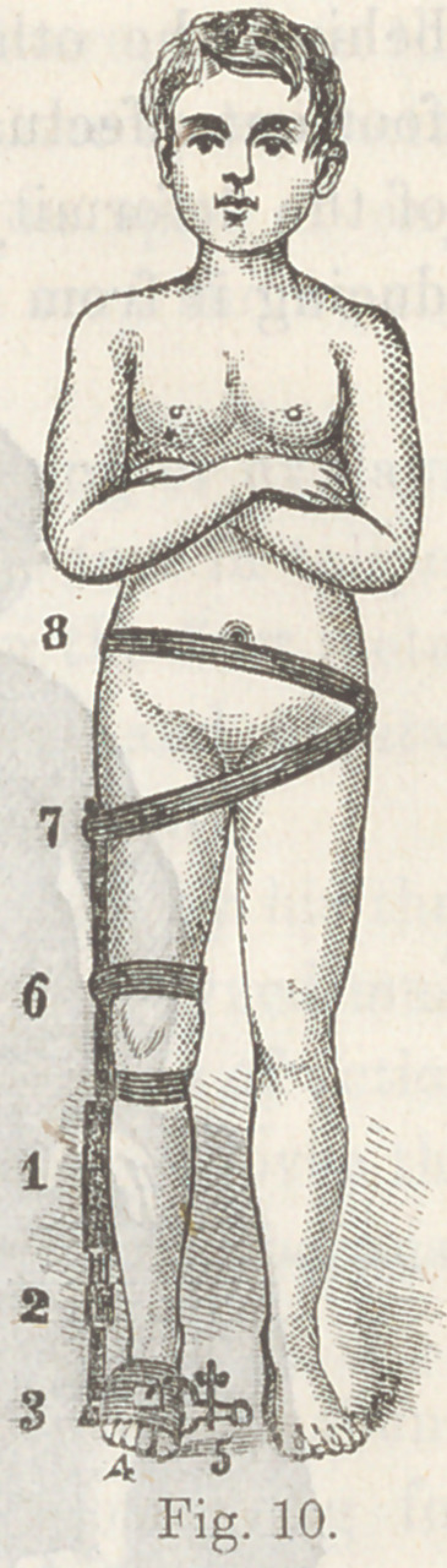


**Fig. 11. f11:**
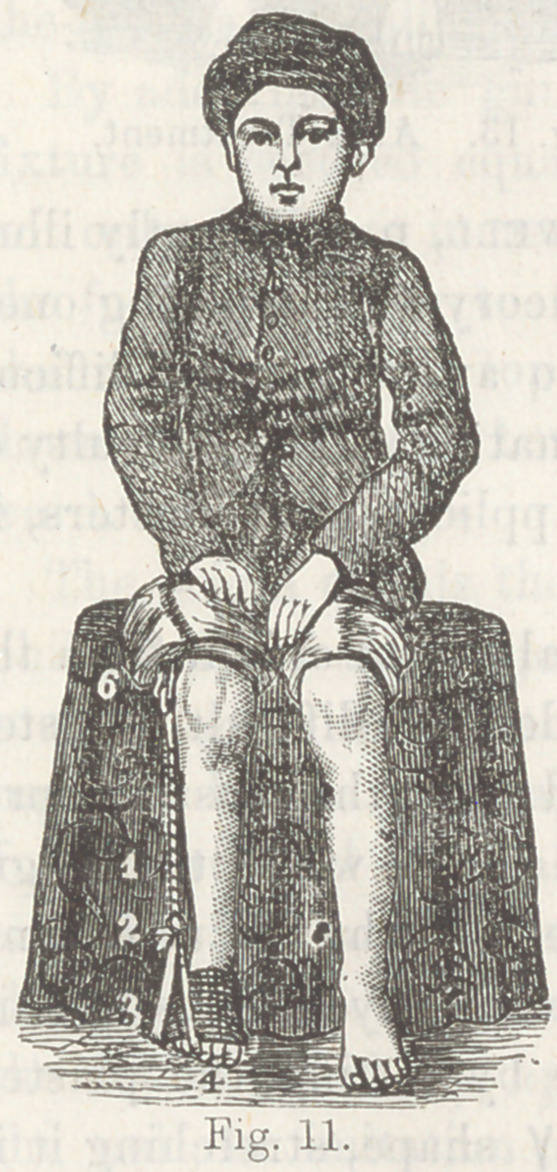


**Fig. 12. f12:**
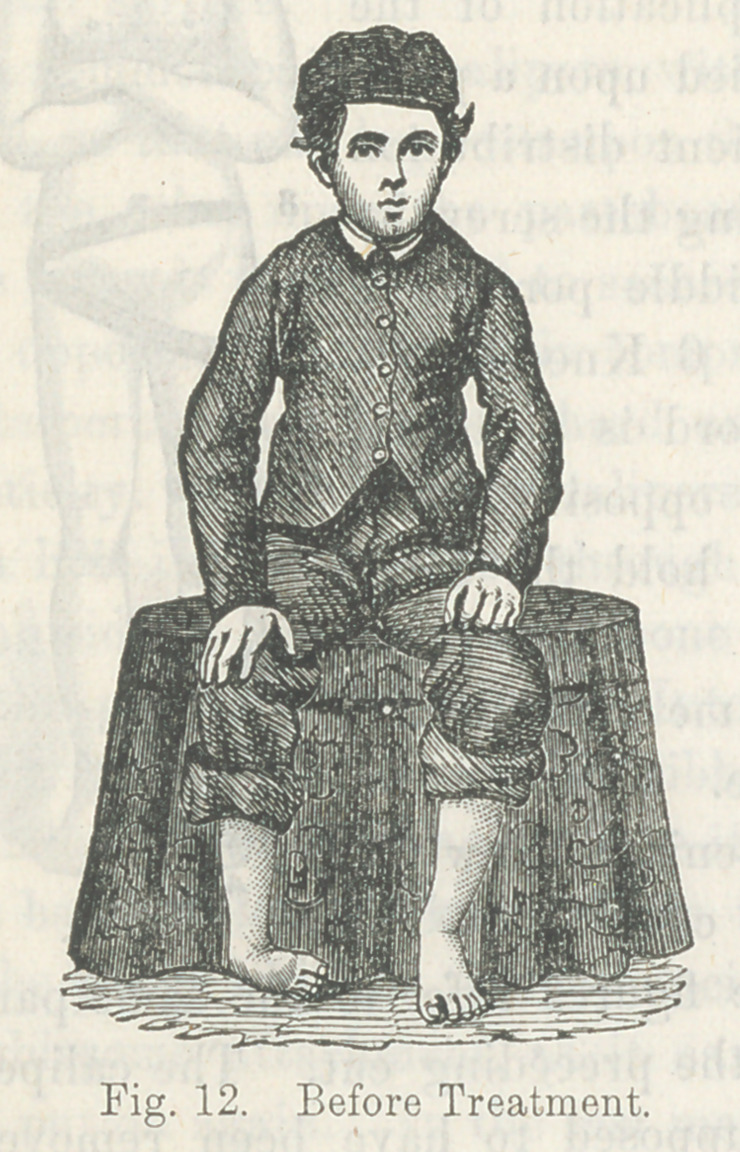


**Fig. 13. f13:**
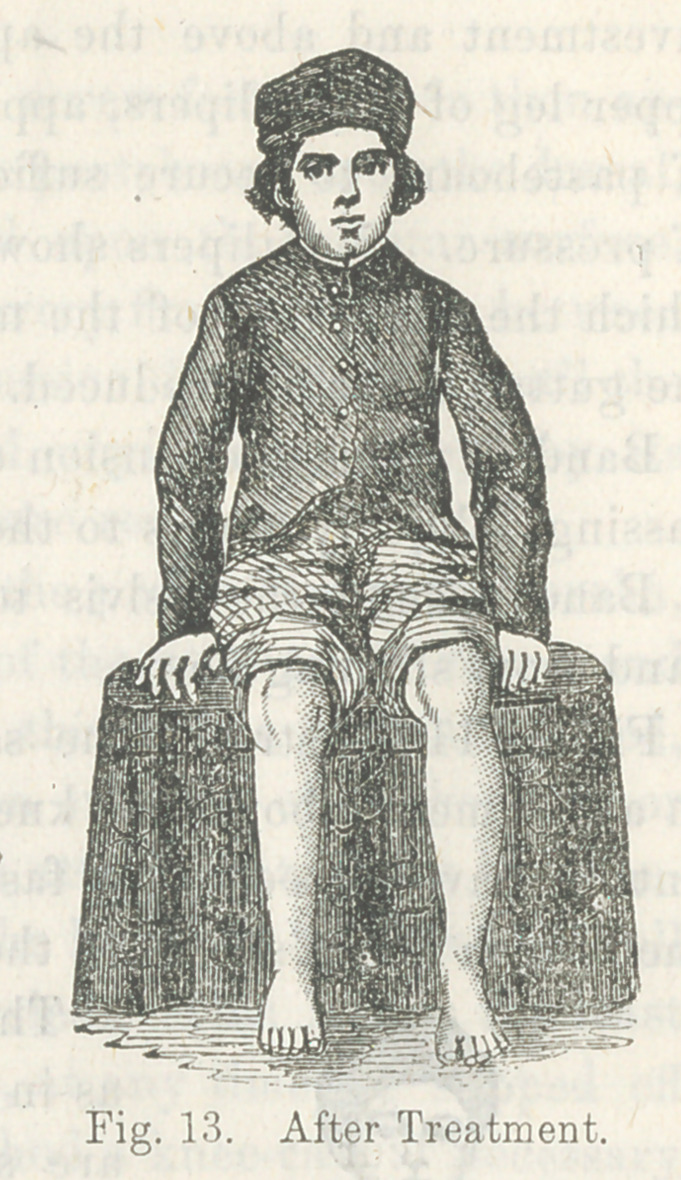


**Fig. 14. f14:**